# Anti-NMDAR encephalitis impairs intrinsic hippocampal dynamics through neuronal hypercoupling, hub dominance, and aberrant ensembles

**DOI:** 10.1038/s41380-026-03568-6

**Published:** 2026-03-31

**Authors:** Vahid Rahmati, Jürgen Graf, Mihai Ceanga, Dario Cuevas Rivera, Holger Haselmann, Sabine Liebscher, Harald Prüss, Knut Holthoff, Knut Kirmse, Christian Geis

**Affiliations:** 1 Section Translational Neuroimmunology, Department of Neurology, Jena University Hospital, 07747 Jena, Germany; 2 Department of Neurology, Jena University Hospital, 07747 Jena, Germany; 3 Department of Psychology, Technical University Dresden, 01187 Dresden, Germany; 4 Institute of Clinical Neuroimmunology, Klinikum der Universität München, Ludwig-Maximilians University Munich, Martinsried, Germany; 5 Institute of Systems Neuroscience, Medical University of Innsbruck, 6020 Innsbruck, Austria; 6 Department of Neurology and Experimental Neurology, Charité - Universitätsmedizin Berlin, Corporate Member of Freie Universität Berlin and Humboldt-Universität Berlin, 10117 Berlin, Germany; 7 German Center for Neurodegenerative Diseases (DZNE) Berlin, 10117 Berlin, Germany; 8 Department of Neurophysiology, Institute of Physiology, University of Würzburg, D-97070 Würzburg, Germany

**Keywords:** Neuroscience, Psychiatric disorders

## Abstract

Autoimmune anti-NMDA-receptor encephalitis is characterized by autoantibody-induced NMDA receptor hypofunction leading to severe neuropsychiatric symptoms including psychosis, hallucinations, memory dysfunction and seizures. However, it remains enigmatic what changes in intrinsic network organization at the multi-neuronal level, serving as the neural substrate of brain function, underlie disease symptomology. Using a mouse model with passive-transfer of patient’s monoclonal anti-GluN1-autoantibodies, we performed two-photon in vivo recordings of spontaneous dynamics under light anesthesia in CA1 microcircuits, a key hippocampal area for memory processing. We find pronounced functional coupling and clustering between putative neurons (PNs), alongside an altered network architecture with pathological emergence of irregular neuronal ensembles. These alterations not only induce excessive hub-like properties but also contribute to the increased network’s intrinsic synchrony, despite its reduced baseline activity; this hypersynchrony was further supported by pathologically faster intra-ripple oscillations and amplified population bursts during these coincident events in vivo. Next, using electrophysiological data ex vivo, we show that this profound functional rewiring is associated with a selective preservation of effectively strong excitatory synapses, despite overall reduced excitation and augmented long-term depression. Furthermore, we find abnormal PN firing characteristics, higher transmission fidelity, and increased similarity of spontaneous spatiotemporal activity patterns, all reflecting dysregulated intrinsic organization of CA1 dynamics. Collectively, the aberrant reorganization of hippocampal microcircuits and altered intrinsic network activity patterns provide new mechanistic insights into the consequences of NMDAR hypofunction and pathomechanisms of anti-NMDAR encephalitis.

## Introduction

NMDA receptors (NMDARs) play a pivotal role in excitatory transmission and both long-term synaptic depression (LTD) and potentiation (LTP), subserving memory, learning, and psychosocial behavior [1–3]. Hypofunction of NMDARs is linked to various neuropsychiatric disorders, such as schizophrenia and Alzheimer’s disease [1, 2, 4, 5]. Anti-NMDAR encephalitis is a severe autoimmune disorder, caused by autoantibodies targeting the NR1 (GluN1-Ab) subunit of the NMDAR [6]. The affected patients share phenotypical similarities to schizophrenia and suffer from severe neuropsychiatric symptoms, ranging from psychosis and cognitive deficits to severe disruption of sleep/wake cycles, dysautonomia, and seizures [6, 7]. The current standard immunotherapy is often insufficient in managing the symptoms in severe cases [7, 8]. GluN1-Ab have been shown to induce NMDA receptor hypofunction by reducing neuronal surface expression of NMDARs due to receptor crosslinking and internalization [9–11], by altered NMDA receptor trafficking in the neuronal membrane and synapse [12–14], and by direct changes in NMDAR neurotransmission [9, 15], finally disrupting NMDAR signaling and NMDA receptor-dependent LTP [9, 10, 16, 17]. Despite the impaired synaptic excitatory signaling [18–20], favoring inhibition dominance, cortico-hippocampal circuits under GluN1-Ab are prone to hypersynchronous discharges [18, 19] and broader altered dynamics [21–24], whose underlying mechanisms remain enigmatic.

Hippocampal networks exhibit a rich repertoire of internally generated activity patterns that are crucial for learning and memory [25–30], processes which are often impaired in anti-NMDAR encephalitis [6, 7]. These spontaneous activity patterns, which are largely independent of sensory stimuli, such as those seen during non-REM sleep, shape internal memory scaffolds, or representational maps, that are integral to these cognitive functions [25–32]. The basis of these patterns relies mainly on functional neuronal coupling (connectivity), mediating both the co-activation and sequential activation of neuronal ensembles, as well as individual neurons within these ensembles [26, 28, 29, 33–36]. Furthermore, alterations in the functional coupling and ensemble dynamics were proposed to underlie brain dysfunction in neuropsychiatric diseases, including schizophrenia [37–39] and epileptic seizures [40, 41]. We hypothesize that impairments in these intrinsic building blocks, which underpin network organization, underlie learning and memory dysfunction in conditions of NMDAR hypofunction, such as anti-NMDAR encephalitis.

Using in vivo Ca^2+^ imaging combined with electrophysiology in a passive-transfer anti-NMDAR encephalitis mouse model [17], we investigate the functional changes in intrinsic hippocampal network dynamics. We find an intricate circuitopathy where overall activity suppression coexists with increased intrinsic synchrony in vivo, corroborated by exaggerated dynamics during concurrently recorded sharp-wave ripples. We identify the circuit-level basis for this duality as a profound network reorganization characterized by higher neuronal coupling, hub dominance, and altered neuronal ensemble properties within a sparser connectivity structure. Mechanistically, we link these alterations to a selective redistribution of excitatory synaptic strength and increased LTD. Collectively, this aberrant functional rewiring and the resulting rigid spatiotemporal dynamics provide new mechanistic insights into the consequences of NMDAR hypofunction and pathomechanisms of anti-NMDAR encephalitis.

## Results

**Fig. 1:**
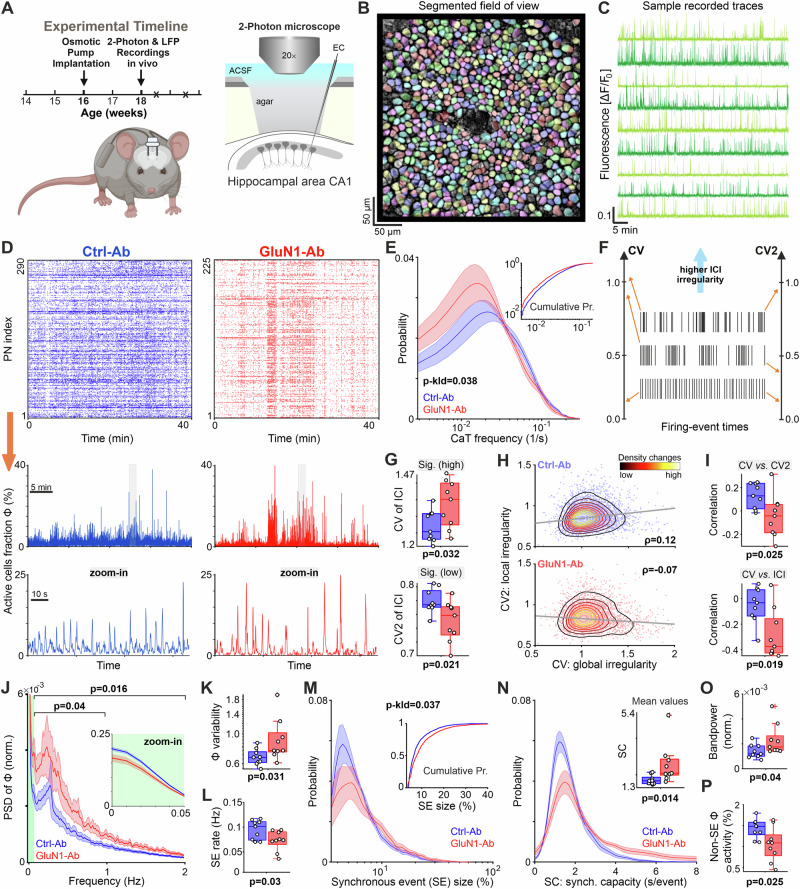
GluN1-Ab increases intrinsic network synchrony while reducing baseline activity in CA1. **A** Schematic of the experimental timeline (left) and recording configurations (right). EC indicates the electrode used for simultaneous extracellular recordings. Created in BioRender; Geis, C. https://BioRender.com/9qs1fwa (2026). **B** Representative field of view (FOV) of 2-Photon recordings from CA1 area using OGB-1-indicator in vivo, overlaid by the detected regions of interest (ROIs) representing the somata of putative neurons (PNs). **C** Timeseries of Ca^2+^ fluctuations extracted from example ROIs, here PNs. **D** Top: rastergrams of reconstructed onsets of PNs’ Ca^2+^ transients (CaT, dots) for two example FOVs (1 FOV per mouse). Middle: the corresponding timeseries of active PNs fraction Φ(t). Bottom: the zoom-in of periods marked in the middle row (gray rectangle). **E** Distributions of mean CaT frequency of PNs. The p-kld denotes the *p*-value of permutation test based on the Kullback-Leibler divergence (KLD) used to compare the overall shape of distributions. Inset: cumulative probability of CaT frequencies of all mice (concatenated per group). **F** Schematic of global (CV) and local irregularity (CV2) measures of inter-CaT intervals (ICIs). Adapted from [52]. **G** Irregularity of PNs with significantly high CV or low CV2 values. **H** Negative relationship between CV and CV2 of ICIs under GluN1-Ab. ρ indicates the average Spearman’s rank correlation coefficient over those computed per mouse. Each dot represents a PN. PNs of all mice are shown. The contour lines indicate the slope of cell-density changes. **I** The relationship between global and local irregularity (top) and median-ICI of PNs (bottom). Spearman’s rank correlation coefficient was computed separately per FOV (i.e. mouse). **J** Power spectral density (PSD) of Φ(t), and corresponding bandpowers. PSD was normalized to total power per mouse. Inset: Zoom-in of PSD in the <0.05 Hz band (green). **K** Variability of network activity fluctuations computed as coefficient of variation of Φ(t). **L** The occurrence rate of synchronous events (SEs). **M** Redistribution of SE size towards larger values under GluN1-Ab. Same format as in (E). **N** Increased synchronization capacity of PNs, i.e. their tendency to participate in SEs, under GluN1-Ab. **O** Bandpower of Φ(t) in the <0.05 Hz band shown in (**J**; green). **P** Median of sub-SE-threshold Φ(t) activity. Curves represent mean ± SEM across mice. Boxplots show median and interquartile range; dots represent individual mice. Sample sizes: (E, G–P) n = 9 mice per group (total cells: 4279 Ctrl-Ab, 3200 GluN1-Ab). Statistical comparisons: two-sample t-tests (**G, I, L, P**), Mann-Whitney U tests (J [bottom], **K, N, O**), or permutation tests (**E, J** [top], **M**); see Supplementary Table 1.

We employed the established passive-transfer mouse model with in vivo chronic intraventricular delivery of pathogenic immunoglobulin G (IgG) via osmotic pumps [17], infusing either NMDAR encephalitis patient-derived monoclonal NMDAR-GluN1 subunit (GluN1-Ab) or control IgG (Ctrl-Ab), for 14–16 days (Fig. 1A, left). We applied acousto-optic two-photon imaging in head-fixed mice to record spontaneous somatic Ca^2+^ transients (CaTs) from individual CA1 putative neurons (PNs) in the *stratum pyramidale*, using the calcium indicator OGB-1 (Fig. 1A, right) [42]. While this layer mainly comprises glutamatergic cells ( > 90%), the recorded population likely includes a small fraction of interneurons [43, 44]. Simultaneously, we performed extracellular local field potential (LFP) recordings from the adjacent, proximal *stratum radiatum* in CA1 (Fig. 1A and Supplementary Fig. 1A,B). To primarily investigate intrinsic activity patterns and network organization while minimizing sensory input [45, 46], experiments were conducted under light anesthesia (isoflurane, ∼0.6%). By reducing external drive, this condition may facilitate the study of internally generated activity sharing features with patterns observed during non-REM/slow-wave sleep (SWS) [36, 45–48]. Consistent with this goal, LFP recordings from both experimental groups were dominated by slow-wave delta-band oscillations (1.5–4 Hz; Supplementary Fig. 1C). Furthermore, the delta band LFP power spectra did not differ between Ctrl-Ab and GluN1-Ab groups (Supplementary Fig. 1C,D), implying a comparable degree of sedation across the groups.

For each mouse, one FOV was recorded for about one hour (57.88 ± 0.91 min, n = 18 mice; mean ± SEM), with a final available duration of 51.73 ± 1.28 min, after excluding residual drifts. Two-photon Ca^2+^ imaging data were subjected afterwards to an offline cell-detection procedure (Fig. 1B). We detected an average of 475.4 ± 32.6 cells per FOV in the Ctrl-Ab group (n = 9 mice; range: 299–629 cells; total: 4279 cells) and 355.6 ± 45.9 cells per FOV in the GluN1-Ab group (n = 9 mice; range: 105–544 cells; total: 3200 cells). For each PN, the fluorescence trace was extracted (Fig. 1C), followed by the reconstruction of CaT onsets, as a proxy for firing activity (Fig. 1D, top) [49]. Each reconstructed event may represent a single-spike or a burst-envelope, since the data temporal resolution (∼100 ms/frame) is longer than the typical intervals of within-burst spikes [42, 49, 50]. Note that somatic calcium imaging using OGB-1, as employed here, primarily detects calcium influx associated with suprathreshold neuronal activity (i.e. spiking activity, rather than synaptic inputs) [50, 51]. In addition, we conducted LTD recordings from CA1 region ex vivo. Full statistical results are provided in Supplementary Table 1.

### GluN1-Ab alters spontaneous firing characteristics of CA1 PNs

First, we investigated GluN1-Ab effects on the firing characteristics (frequency and structure) of CA1-PNs (Fig. 1D–I). This analysis revealed an overall alteration in the spontaneous CaT frequency distribution, with an increase in number of neurons with lower activity rather than a mean shift across the entire spectrum (Fig. 1E, Supplementary Table 1). We then, for each neuron, assessed the regularity of its firing pattern using the coefficient of variation (CV) for *global* irregularity, which quantifies the *overall* variability of inter-CaT intervals (ICIs) across the entire recording relative to the mean interval. Additionally, we calculated CV2 for *local* irregularity, which quantifies the variability specifically between consecutive ICIs [52]. GluN1-Ab tended to increase the global irregularity (CV) but to diminish local irregularity (CV2) of ICIs (Fig. 1F–I, Supplementary Fig. 2A–D). To assess the significance of each PN’s CV and CV2, we compared them against population-level irregularity thresholds (Thr_Low_ and Thr_High_) derived from randomized surrogate event trains generated for each neuron (see Supplementary Fig. 2C for details). PNs with a significantly high CV showed even higher CV levels, while PNs with a significantly low CV2 exhibited even lower CV2 levels under GluN1-Ab (Fig. 1G, Supplementary Fig. 2D). This alteration was also reflected in the correlation between CV and CV2 of ICIs, which reduced from positive under Ctrl-Ab to negative values under GluN1-Ab (Fig. 1H,I). These observations suggest sporadically structured (or burst-like) activity of individual PNs at relatively short timescales ( < 1 s; see below) under GluN1-Ab (Fig. 1D, top). This was further corroborated by several findings, including the reduced correlation between the CV and median of ICIs (Fig. 1I; Supplementary Fig. 2E) and the higher fraction of PNs having positive correlation between successive ICIs (Supplementary Fig. 2F).

### GluN1-Ab increases PN recruitment during spontaneous synchronous events despite reduced network activity

Hippocampal activity consists of a dynamical interplay between irregular and rhythmic neuronal activity patterns. Therefore, we next investigated the influence of altered firing properties on the intrinsic dynamics of network activity. To this end, we analyzed Φ(t) as the fraction of spontaneously active PNs across time (Fig. 1D, middle; Supplementary Fig. 2G). The power spectrum of Φ(t) primarily increased over the 0.05–2 Hz range (Fig. 1J), encompassing the distinct peak (∼0.05–0.5 Hz) present in both groups, which suggests excessive coherency of PNs’ activities under GluN1-Ab. This was corroborated by the heightened variability of Φ(t) under GluN1-Ab (Fig. 1K), implying sporadic surges in network activity (Fig. 1D). We next assessed how GluN1-Ab affects spontaneous synchronous events (SE), extracted as those activity periods in Φ(t) which significantly surpass detection threshold, determined separately for each FOV by randomizing CaT events per PN [42]. We found a decrease in SE occurrence rate (Fig. 1L) without a change in the detection threshold or the duration of SEs (Supplementary Fig. 2H,I). However, SE size (i.e. the fraction of PNs activated per SE) increased (Fig. 1M), and individual PNs were more frequently engaged in SEs under GluN1-Ab (Fig. 1N). Note that these SEs represent substantial population co-activations (mean of the minimum SE sizes: 18.5 ± 1.3 cells), with an absolute minimum size of 9 cells observed across all mice of both groups (n = 18 mice). Strikingly, this increased synchrony by GluN1-Ab was despite the reduction of Φ(t) power in <0.05 Hz band (Fig. 1J,O) and of non-synchronous activity (Fig. 1P), both indicative of lowered baseline network activity (see also Supplementary Fig. 2G). Together, these results imply that GluN1-Ab reduces baseline spontaneous network activity while simultaneously enhancing the intrinsic synchronization of PNs, reflecting excessive network synchrony.

### GluN1-Ab induces intrinsic hypercoupling of CA1 PNs

**Fig. 2:**
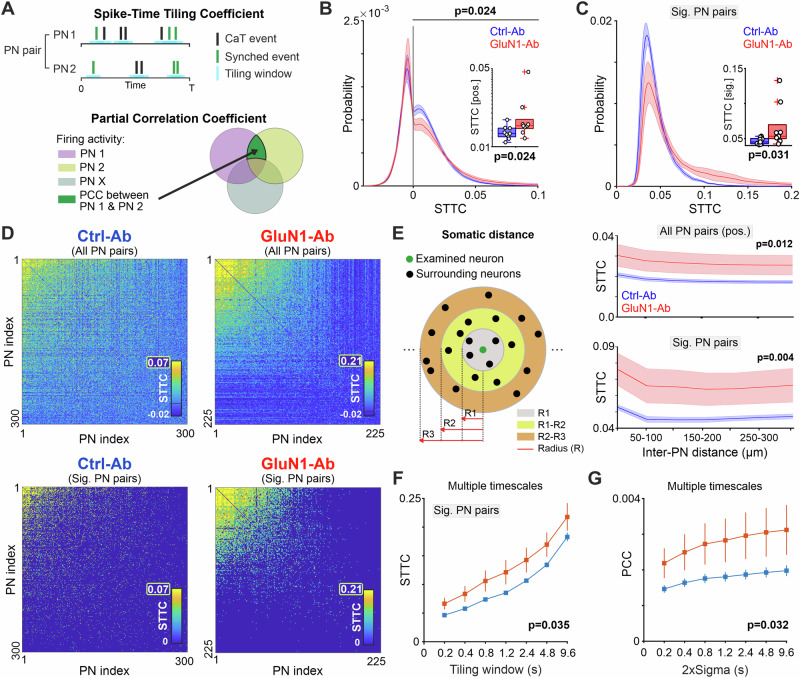
GluN1-Ab induces intrinsic hypercoupling of CA1 PNs. **A** Schematic representation of the STTC and PCC quantifications of coupling between CaT-trains of putative neuron (PN) pairs. **B** Distribution of STTC indices, considering all PN pairs (tiling window of 0.2 sec). For boxplots, only pairs with positive (pos.) coupling were considered. **C** Increased coupling of PN pairs with significantly high STTC (see Supplementary Fig. 3A,B) under GluN1-Ab. Same format as in (**B**). **D** Example STTC matrices (re-ordered) of all (top row) and significantly (bottom row) coupled PN pairs from two individual FOVs. **E** Relationship between STTC and Euclidean somatic distance of PNs. Left, schematic of inter-PN distance used for quantification of the relationships shown in the right panels. For example, for 50–100 μm, for each PN, its average coupling with neurons locating within this range are computed. Radius: ca. 50 to 350 μm. (**F, G**) Increased neuronal coupling under GluN1-Ab at various timescales. The measures were quantified at multiple tiling windows of STTC (**F**) or standard deviation of Gaussian kernel used for smoothing CaT-trains in PCC (**G**). Curves represent mean ± SEM across mice. Boxplots show median and interquartile range; dots represent individual mice. Sample sizes: (**B–C, E–G**) n = 9 mice per group (total cells: 4279 Ctrl-Ab, 3200 GluN1-Ab). Statistical comparisons: Mann-Whitney U tests (**B, C**) or permutation tests (**E–G**); see Supplementary Table 1.

We hypothesized that alterations in pairwise functional coupling might underlie enhanced synchrony under GluN1-Ab, despite overall reduced activity levels. Therefore, we used the spike-time tiling coefficient (STTC), as measure of pairwise functional coupling largely independent of overall firing rates [53], to quantify, for each PN pair, the degree of coordinated activity between their CaT event trains within specific short-time tiling windows around CaT events (Fig. 2A–F). STTC was higher in GluN1-Ab mice, particularly in positively correlated PN pairs (Figs. 2B, 2D top). This effect was more evident in pairs with significantly higher-than-chance correlation (Figs. 2C, 2D bottom and Supplementary Fig. 3A,B), although the fraction of such pairs ( < 10%) remained unchanged (Supplementary Fig. 3C). Analyzing STTC relative to the inter-PN distance revealed increased coupling between both nearby PNs and those farther apart (Fig. 2E). We next assessed the dependency of this effect on the temporal range considered for measuring the correlation (tiling window). This analysis showed a higher coupling of the significant pairs under GluN1-Ab over a relatively broad range of timescales (Fig. 2F, Supplementary Fig. 3D), suggesting an alteration of intrinsic neural timescales of information processing [54, 55]. To further assess the robustness of our findings, we also calculated the Pearson correlation coefficient (ρCC) between Gaussian-smoothed CaT event-trains of PN pairs [42, 56, 57]. This standard measure of functional coupling yielded qualitatively consistent results (Supplementary Fig. 3E,F). Of note, both STTC and ρCC can be influenced by the activity of other neurons, potentially mediating indirect relationships. Hence, as a measure attempting to isolate direct pairwise coupling, we utilized the partial correlation coefficient (PCC; Fig. 2A) [58, 59]. Technically, PCC extends ρCC by statistically accounting for (i.e. regressing out) the linear influence of all other neurons when calculating the correlation between a specific neuron pair. The PCC coupling results (Fig. 2G, Supplementary Fig. 3G) were qualitatively similar to those obtained using STTC and ρCC. Finally, our simulated results, based on bursting neuron models [50], showed that these measures retain their sensitivity to relative variations in coupling strength, despite the relatively low temporal resolution (Supplementary Fig. 3H–K). Together, our data suggest that the higher intrinsic functional coupling (neuronal hypercoupling) of a subset of CA1-PNs, observed across extended spatial and temporal scales, contributes to the increased network synchrony under GluN1-Ab.

### Aberrant functional rewiring and neuronal ensembles in CA1 network under GluN1-Ab

**Fig. 3:**
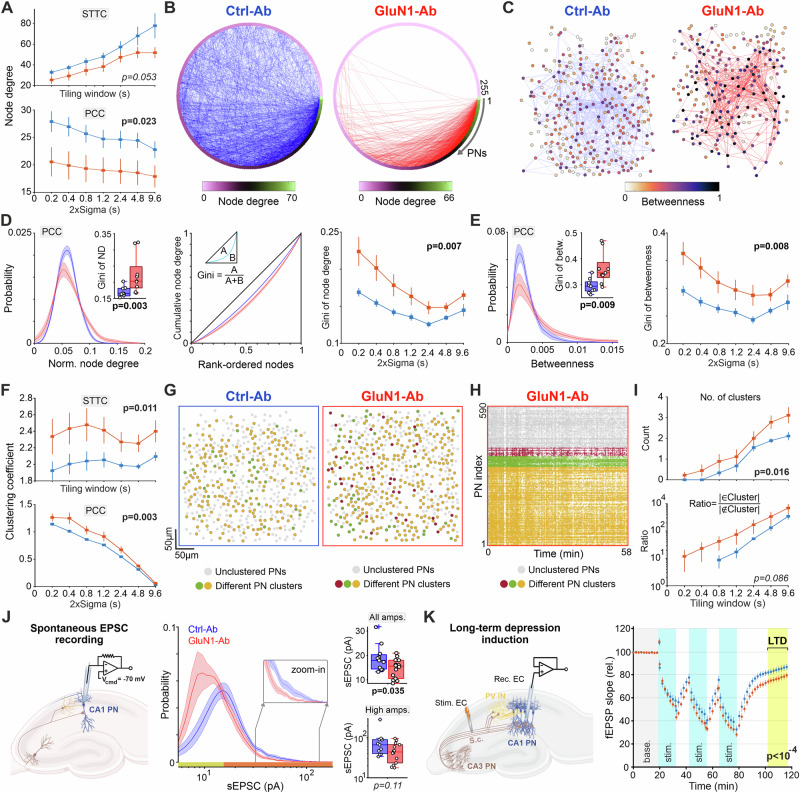
Aberrant functional rewiring and neuronal ensembles alongside globally reduced connectivity links in CA1-network under GluN1-Ab. **A** Overall reduction in node degree under GluN1-Ab over different timescales, quantified based on STTC (top) and PCC (bottom) indices. Same format as in Fig. 2F,G. **B** Circular graphs with links (connections) and nodes (putative neurons [PNs]) of two example FOVs. Nodes were ordered in descending manner based on the ND of PNs, and links (unweighted) were drawn based on STTC matrices (tiling window of 0.2 s). To ease visualization, only links with a STTC above 95^th^ percentile (separately for each FOV) were shown. **C** Functional connectivity patterns of example graphs in (**B**). Each dot is a color-coded node (PN) based on its betweenness centrality. To ease visualization of hub-like PNs (darker dots), the values were normalized to the maximum betweenness of the two FOVs. Line thickness encodes the STTC-based connection strengths, normalized to maximum STTC for each FOV separately. **D** Distribution of node degree normalized to the number of nodes per network (left, tiling window of 0.2 s), its Lorenz curve (middle), and the corresponding Gini coefficients at different timescale. Lorenz curves demonstrate the cumulative proportion of node degrees against the cumulative proportion of PNs rank-ordered by their node degree. The line of equality represents the case where all PNs have equal node degrees. Gini quantifies the deviation from equality. **E** Similarly to (**D**), but for betweenness. **F** Increased clustering coefficient of PNs under GluN1-Ab. **G** Example FOVs with detected PN ensembles. For clarity, ensemble overlaps were not shown. **H** Rastergram of the FOV shown in (**G**) under GluN1-Ab, with color-coded and re-arranged CaT-trains of PNs based on their affiliated ensemble. **I** Number of detected PN ensembles (top) and the ratio of PNs in ensembles to unaffiliated PNs (bottom), per FOV. **J** Intracellular recording of sEPSCs from CA1-PNs in hippocampal slices. Left, schematic of recording configuration. Created in BioRender; Geis, C. https://BioRender.com/9qs1fwa (2026). Middle, distribution of sEPSC amplitude. Right, the median of all sEPSC amplitudes (top) and those above 95^th^ percentile (bottom), computed separately per neuron. **K** Stronger long-term depression (LTD) under GluN1-Ab. Left, schematic of recording configuration. Created in BioRender; Geis, C. https://BioRender.com/9qs1fwa (2026). Right, slope of field EPSP relative to that of baseline period (gray). Yellow-colored period was analyzed for between-group difference after LTD induction. Curves represent mean ± SEM. Boxplots show median and interquartile range; dots represent individual mice, cells, or slices. Sample sizes: (**A, D–F, I**) n = 9 mice/group (total cells: 4279 Ctrl-Ab, 3200 GluN1-Ab); (J) n = 11 (Ctrl-Ab) / 14 (GluN1-Ab) cells; (K) n = 16 (Ctrl-Ab) / 21 (GluN1-Ab) slices. Statistical comparisons: two-sample t-tests (**E** [left], **J** [top]), Mann-Whitney U tests (**D** [left], **J** [bottom]), permutation tests (**A, D** [right], E [right], **F, I**), or mixed-model analysis (**K**); see Supplementary Table 1.

How does altered neuronal coupling (Fig. 2) influence the intrinsic functional organization of CA1 microcircuits? Using complex network analysis [60], we represented PNs as nodes in a graph, with edges defined by coupling strength (STTC or PCC). Absolute node degree, the raw count of relatively strong connections per PN, decreased under GluN1-Ab (Fig. 3A–C), reflecting a broad reduction in functional connectivity of the network overall (Supplementary Fig. 4A). In contrast, the normalized node degree, which adjusts for network size, showed a stable mean across groups (Supplementary Fig. 4B). However, the Gini coefficient (measuring distribution inequality) [61] and the hub-enrichment ratio (75^th^/50^th^ percentile, highlighting upper-tail prominence), the two normalization-independent hubness metrics, both increased (Fig. 3D and Supplementary Fig. 4C,D). This implies that the remaining connectivity is concentrated on a subset of PNs with disproportionately high connectivity under GluN1-Ab. Beyond node degree, betweenness centrality, which quantifies a PN’s role as a crucial intermediary along the shortest path between other PNs, also revealed excessive hubness (Fig. 3E and Supplementary Fig. 4C,E). Under GluN1-Ab, its mean, Gini coefficient, and hub enrichment ratio increased, suggesting a greater prevalence of pivotal nodes that mediate network communication. Additionally, several widely-used centrality measures showed higher levels (Fig. 3F and Supplementary Fig. 4F), including the clustering coefficient which indicates increased prevalence of closed triangular motifs (three fully interconnected PNs) under GluN1-Ab (Fig. 3F). These findings collectively imply that the overall reduced connectivity links is accompanied by both pathologically higher hub-like properties and neuronal clustering in the functional backbone of PN-network under GluN1-Ab (for examples see Fig. 3B,C). To investigate whether this altered topology reflect changes in neuronal ensembles, we next performed an ensemble detection analysis [42, 62] using STTC, chosen for its robustness in capturing both direct and indirect coupling of PNs. The number of intrinsic functional ensembles was higher in the GluN1-Ab group, whereas the total number of PNs assigned to ensembles relative to those unaffiliated PNs remained unchanged (Fig. 3G–I, Supplementary Fig. 4G). Similar results were obtained using ρCC for ensemble detection (Supplementary Fig. 4H), showing their robustness across coupling measures.

These results suggest that the PNs’ hypercoupling under GluN1-Ab is accompanied by an aberrant functional rewiring of intrinsic CA1 circuity with more functional PN-ensembles, characterized by excessive network hubness and clustering without an increase in number of functional connections.

### Selective maintenance of effectively strong excitatory synapses and augmented LTD under GluN1-Ab

How can the higher functional coupling and clustering observed here be reconciled with the previous reports about GluN1-Ab-induced overall decrease in excitatory synaptic strength onto CA1-PNs ex vivo [18] and the well-established disrupted LTP [6, 17]? Re-analysis of our previously published spontaneous EPSCs data ex vivo [18] supports the hypothesis that selectively maintained strong excitatory synapses provide the basis for the remaining, strong functional links: despite an overall reduction in the EPSC amplitude under GluN1-Ab, the range of the distribution’s right tail, representing strong synaptic connections, remained largely preserved (Fig. 3J).

Alongside the excessive functional coupling, however, the overall network connectivity was reduced (Fig. 3A and Supplementary Fig. 4A) and the network organization partitioned into a higher number of neuronal ensembles (Fig. 3G-I), indicating that widespread synaptic weakening or elimination also occurs. Given that NMDAR mediate certain forms of LTD [1–3], we hypothesized that GluN1-Ab abnormally facilitates this process. Such augmented LTD, reported for CA1 in other neurological disorders [63, 64], could selectively filter out weaker functional connections [1–3], which could potentially be crucial for integrating activity across the broader network and maintaining the stability of larger ensemble structures [34, 65–67]. To test this potentially crucial and less-understood role of LTD in the observed functional network pathology, we performed recordings in hippocampal slices ex vivo using an NMDAR-dependent LTD induction protocol at Schaffer-Collateral synapses onto CA1-PNs [68] (Fig. 3K, left). While the LTD protocol induced ∼20% reduction in synaptic strength under Ctrl-Ab, the depression was significantly stronger (by a further ∼10%) under GluN1-Ab (Fig. 3K). This finding suggests that the augmented NMDAR-dependent LTD might have contributed to functional network pathology by pruning weaker functional connections, leading both to an overall reduction in functional connections (Fig. 3A and Supplementary Fig. 4A) and emergence of more numerous neuronal ensembles under GluN1-Ab (Fig. 3G–I, Supplementary Fig. 4G,H). Furthermore, such preferential elimination of weaker connections while preserving the stronger connections could potentially increase the relative prominence of remaining strong connections, thereby contributing to the observed increase in the network hubness (Fig. 3D,E and Supplementary Fig. 4C–F).

### GluN1-Ab leads to higher transmission fidelity and restructures CA1 spatiotemporal activity patterns around aberrant ensembles

**Fig. 4:**
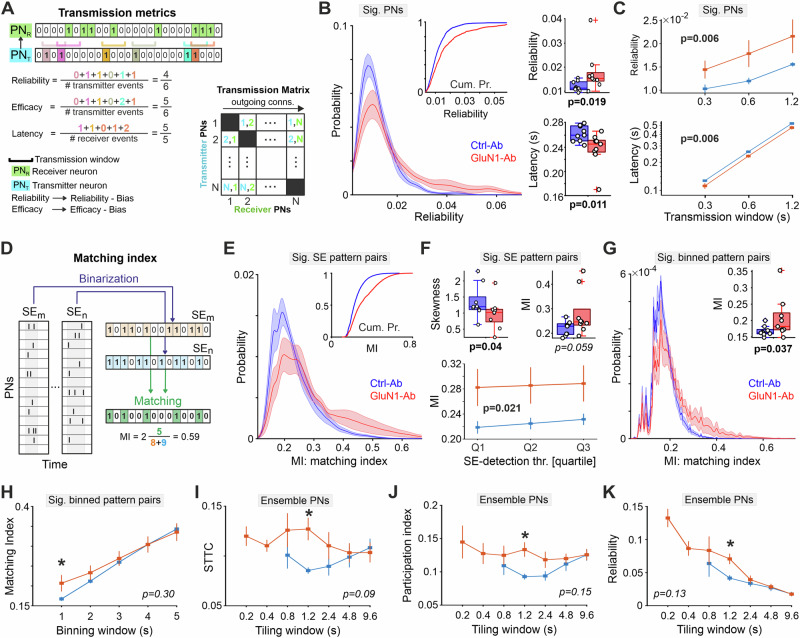
GluN1-Ab leads to higher transmission fidelity and restructures CA1 spatiotemporal activity patterns around aberrant ensembles. **A** Schematic representation of inter-neuronal transmission metrics for a single potential transmitter (PN_T_) to a potential receiver (PN_R_) based on the CaT-onset trains of putative neurons (PNs). Reliability: fraction of successfully transmitted CaTs; Latency: average time delay of this transmission; Efficacy: average number of CaTs transmitted to PN_R_, upon PN_T_ activation. Bias denotes the randomization-based value of each metric. For each PN_T_, its value for a given metric is determined as the mean or median over its outgoing connections (to PN_R_s), i.e. over its corresponding row in the depicted transmission matrix. **B** Higher reliability and reduced latency of individual PNs’ transmission to others. Per metric, PN_T_s with significant level (e.g. of reliability), as compared to randomized data, were considered (sig. PNs). Transmission window was set to 0.6 s. **C** Transmission metrics of sig. PNs at different plausible timescales. **D** Schematic representation of similarity quantification between a pair of SE patterns using the matching index (MI). **E, F** Excessive similarity between SE spatial-pattern pairs with significantly high MIs, compared to similarity between spatially randomized SE patterns. **E** Distribution of significant MIs. Same format as in Fig. 1E. **F** Top, boxplots of summary statistics of significant MIs. Bottom, significant MIs as a function of minimum fraction of active PNs in each SE pattern. Quartiles of the SE-detection thresholds of all mice: Q1 ≈ 3.9, Q2 ≈ 4.3, Q3 ≈ 4.9%. **G** Excessive similarity between the spatial patterns, obtained by a non-overlap binning of entire recording time using a relatively short, fixed bin-size (∼1 s). The binned pattern pairs with significantly high MIs were shown, similarly to (**E**). Inset: boxplot of corresponding median values. **H** Similarly to inset in (**G**), but as a function of bin-size. (**I–K**) Functional and transmission metrics of PNs belonging to the neuronal ensembles in Fig. 3G–I, analyzed at various timescales. Same format as in Fig. 2F. Transmission window was set to 1.2 s in (**K**). Curves represent mean ± SEM across mice. Boxplots show median and interquartile range; dots represent individual mice. Sample sizes: (**B, C, E–K**) n = 9 mice/group (total cells: 4279 Ctrl-Ab, 3200 GluN1-Ab). Statistical comparisons: two-sample t-tests (asterisks in I–K), Mann-Whitney U tests (**B, F**[top], **G**, asterisk in H), or permutation tests (**C, F** [bottom], **H-K**); see Supplementary Table 1.

How do these altered functional and activity properties affect inter-neuronal transmission and the spatiotemporal structure of spontaneous activity patterns in CA1? We started by investigating GluN1-Ab effect on inter-neuronal transmission [69–71], by considering each PN as a transmitter (PN_T_) and any other PN as a potential receiver (PN_R_) (Fig. 4A). We then quantified the reliability, latency, and efficacy of CaT transmission for each pair (PN_T_→PN_R_) within a determined transmission time-window (Fig. 4A), followed by assessing their statistical significance using randomized data (Supplementary Fig. 3A). The PNs involved in the significant transmission relationships exhibited higher reliability and efficacy with shorter latency under GluN1-Ab (Fig. 4B–C, Supplementary Fig. 5A–B), although the fraction of such PNs remained relatively low and unchanged (Supplementary Fig. 5C). Together, these findings indicate a selective higher transmission fidelity between PNs under GluN1-Ab.

We next examined the intrinsic structure and recurrence of network-level activity patterns. We first quantified the overlap between spatial patterns of SEs using matching index (MI; Fig. 4D) and extracted significant MI values by comparing to randomized patterns [42, 72]. Despite reducing SE rate (Fig. 1L), GluN1-Ab skewed the distribution of significant MIs towards higher values, alongside a robustly higher MI across different thresholds, used to set the minimum fraction of active PNs per pattern (Fig. 4E–F, Supplementary Fig. 5D). Moreover, to assess whether this aberrant pattern stereotypy is not limited to detected SEs, we repeated the analysis using the spatial patterns obtained by dividing the entire recording time into non-overlapping bins. Pattern similarity was again higher under GluN1-Ab (Fig. 4G), but only with a bin-size covering the range of SE durations (∼1 s, Supplementary Fig. 2I) and not with longer bins (Fig. 4H). This raises the question of whether this abnormality is linked to the pathological characteristics of neuronal ensembles (Fig. 3G–I). Strikingly, ensemble PNs exhibited higher functional coupling and a stronger contribution to their respective ensembles under GluN1-Ab, specifically around the timescale of SEs (Fig. 4I–J, Supplementary Fig. 5F). A similar effect was also observed in their transmission fidelity (Fig. 4K, Supplementary Fig. 5G). These results indicate that the intrinsic organization of internally generated patterns is primarily dysregulated by aberrant neuronal ensembles, resulting in rigid and stereotyped activity patterns.

### GluN1-Ab induces faster ripples and amplifies SPW-R-associated synchronous events

Finally, we investigated sharp-wave ripple (SPW-R) events, which are crucial for memory consolidation and retrieval [73, 74], and represent the electrophysiological hallmark of internally-generated, highly synchronous neural firing, most prevalent during non-REM sleep [73–75]. Importantly, these events operate on a notably shorter timescale ( < 100 ms) compared to the considerably slower population bursts detected as SEs in our two-photon imaging data (ca. 400–1000 ms; Supplementary Fig. 2I). Considering the profound changes in the intrinsic functional network connectivity and activity patterns (Figs. 1–4), we hypothesized an increase in SPW-R amplitude and/or frequency. To this end, we characterized SPW-R properties using our simultaneously recorded LFP data (Supplementary Fig. 1), where SPW-Rs were identified as transient high-power events in the ripple band (80–250 Hz, a range accommodating potential shifts in ripple frequency under anesthesia [76]; see also [75, 77]), coinciding with a sharp-wave component characterized by a large-amplitude deflection in the LFP (5–40 Hz) [75, 77] (Supplementary Fig. 6A).

We found that the mean intra-ripple frequency, derived from the inter-peak intervals of the oscillations per SPW-R event, was higher under GluN1-Ab (Supplementary Fig. 6B). Consistently, the maximum instantaneous frequency achieved during ripples was also increased, indicating that the network oscillates with a pathologically accelerated rhythm in the ripple band. Despite this acceleration in ripple rhythm, the overall occurrence rate or duration of these events remained unchanged (Supplementary Fig. 6C,D), and there was no difference between the groups in the amplitude of neither ripples (Supplementary Fig. 6E) nor sharp-wave components of SPW-R complexes (Supplementary Fig. 6F).

Motivated by these findings, we next asked whether the faster oscillations of SPW-Rs translate to an amplified recruitment of the neuronal population. To address this, we temporally aligned the LFP and two-photon imaging datasets and classified SPW-Rs based on their coincidence with an SE ( ± 300 ms window). We found that in both groups, a similar proportion (approx. 40%) of SPW-Rs coincided with SEs (Supplementary Fig. 6G). However, the population activity Φ(t) specifically during these coincident [( + SE)] events was profoundly higher in GluN1-Ab mice (Supplementary Fig. 6H–J). This indicates that while the propensity for SPW-R/SE coincidence is preserved, the resulting synchronous discharge is pathologically amplified.

In sum, the abnormally faster intra-ripple frequency and the amplified population recruitment during the coincident events jointly point to the susceptibility of CA1 to a hypersynchronous state under GluN1-Ab, consistent with the higher neuronal coupling, clustering and larger synchronous events observed in our imaging data.

## Discussion

**Fig. 5:**
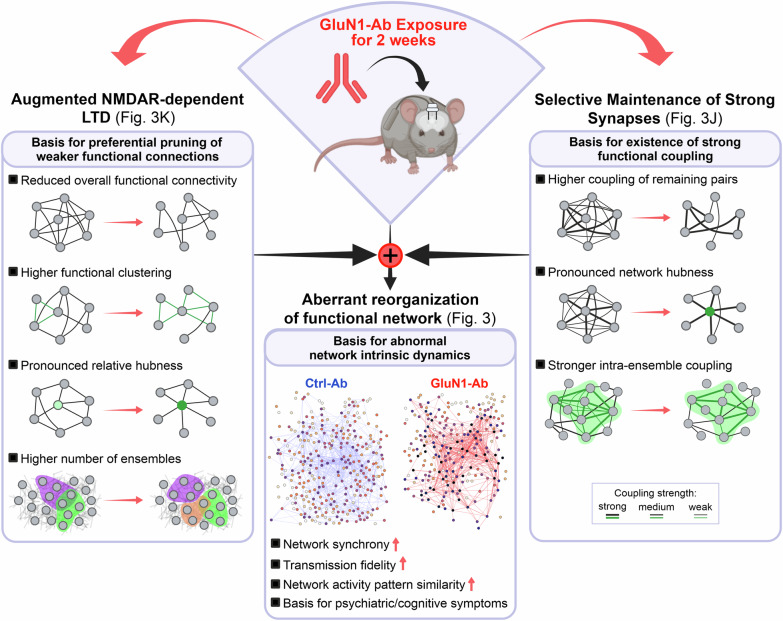
Schematic illustrating proposed mechanism linking synaptic alterations to functional network reorganization and dynamics under GluN1-Ab. We propose two concurrent processes drive the observed circuitopathy: (**Left**) Augmented NMDAR-dependent LTD (Fig. 3K) preferentially filters out weaker functional connections. This selective pruning leads to a sparser connectivity landscape (reduced overall functional connectivity; Fig. 3A, Supplementary Fig. 4A) and the partitioning of network organization into more numerous ensembles (Fig. 3G–I). Crucially, this removal of relatively weaker links increases the relative prominence of remaining local structures and stronger nodes, thereby contributing to higher functional clustering (Fig. 3F) and relative network hubness (Fig. 3D,E). (**Right**) Simultaneously, the selective maintenance of stronger excitatory synapses (supported by Fig. 3J) provides a backbone for presence of strong functional coupling between specific putative neuron (PN) pairs (Fig. 2). In the resulting sparser connectivity landscape (see Left), this maintenance manifests as relatively higher coupling (Fig. 2), network hubness (Fig. 3D,E), and intra-ensemble coupling (Fig. 4I). **(Synthesis)** The proposed interplay of these processes drives the GluN1-Ab-induced functional network rewiring observed in vivo, resulting in higher network synchrony (Fig. 1), inter-neuronal transmission fidelity (Fig. 4A–C,K), and similarity of spatiotemporal activity patterns (Fig. 4D–H). These profound alterations may contribute to the psychiatric and cognitive symptoms in NMDAR-Ab encephalitis (see Discussion). The mouse graphic was created in BioRender; Geis, C. https://BioRender.com/9qs1fwa (2026).

This study aimed to uncover changes in hippocampal network organization and dynamics as a potential substrate for the cognitive, behavioral, and psychiatric disease symptoms in anti-NMDAR encephalitis. Here, using an established anti-NMDAR encephalitis mouse model, we provided new mechanistic insights by investigating how long-term exposure to GluN1-Ab alters intrinsic activity patterns in CA1 under light anesthesia without external sensory stimulation. Our data reveal a specific functional network pathology: GluN1-Ab reduces both overall, asynchronous network activity, and the frequency of synchronous events, rendering PN-network generally hypoactive. Simultaneously, the network undergoes a significant functional rewiring (Fig. 5), characterized by pronounced neuronal coupling, clustering, and hubness, and emergence of more neuronal ensembles, despite overall reduced functional connectivity (number of links). These findings are potentially explained by an interplay between the selective maintenance of strong excitatory synapses and the augmented LTD (Fig. 5, see below for details). Collectively, this aberrant reorganization results in altered intrinsic network dynamics manifesting as increased network synchrony, faster intra-ripple oscillations with exaggerated population recruitment, higher transmission fidelity, and a reduced, atypical repertoire of spatiotemporal patterns. These indicate that anti-NMDAR encephalitis profoundly alters the intrinsic organization and dynamics of hippocampal output microcircuits.

Next, we address the question of how the network exhibits an intrinsic state favoring hypersynchrony, while baseline network activity is suppressed under GluN1-Ab. Firstly, this suppression likely arises from the reduced excitatory currents, as previously shown before for NMDAR currents in in vitro and ex vivo models [9, 10, 16, 17, 20], but also for AMPAR mediated currents [18, 19, 78], using patient-derived IgG and monoclonal antibodies including IgG 003–102. These reduction in excitatory drive induces an E-I-imbalance towards inhibition dominance [18, 19] (see also [21]). In particular, the possible CA1-PN hypoactivity aligns with recent ex vivo reports under GluN1-Ab, showing reduced excitatory synaptic input onto CA1-PNs; e.g. through CA3→CA1 [18]. Secondly, corresponding theoretical models proposed that disinhibition of neural networks [18] or their exposure to instability and pathological dynamics [18, 19, 21] underlie the induced network hypersynchrony. Beyond these modeling predictions, our empirical data unveiled a new mechanism: GluN1-Ab leads to pronounced functional coupling and clustering among PNs as well as higher network hubness. Together, these alterations abnormally facilitate the recruitment of neurons into coordinated network activities, despite reduced baseline network activity and overall functional connectivity. This aberrant functional rewiring can render the neural networks intrinsically susceptible to epileptic seizures [40, 41] and disturb synaptic plasticity processes which, in turn, may underlie severe learning and memory deficits of patients with anti-NMDAR encephalitis [6, 7]. Moreover, similar coexistence of low network activity and relatively large synchrony have been observed e.g. in several studies of SYNGAP1 [79, 80], Alzheimer’s disease [81, 82], PCDH19-epilepsy syndrome [83, 84], and in immature networks [42, 56, 85].

This intrinsic susceptibility to hypersynchrony is further corroborated by our analysis of SPW-Rs. We found an increased intra-ripple frequency, which aligns with our previous finding that GluN1-Abs accelerate GABAergic decay kinetics ex vivo [18]. Since local feedback inhibition kinetics play an important role in pacing the ripple frequency [74, 76, 86, 87], this faster synaptic inhibition theoretically supports the observed oscillation acceleration [88]. Crucially, this hypersynchronous state extends beyond the ripple’s internal rhythm: while the coincidence rate of SPW-Rs and SEs was preserved, the resulting population bursts during these coupled events were starkly amplified. This suggests that the entrainment between SPW-Rs and SEs occurs within a functionally hyper-coupled network that is primed for excessive recruitment. Notably, such aberrant SPW-R dynamics have been reported in animal models of other neuropsychiatric disorders characterized by network instability and memory deficits, including early-stage Alzheimer’s disease [89] (or dementia [90]), schizophrenia [91, 92], and temporal lobe epilepsy [93, 94] (see also [73]). Together with our observed excessive functional coupling and clustering, these alterations—faster ripples and amplified population recruitment—may serve as a distinct electrophysiological signature of the pervasive ‘hypersynchrony-prone’ network state induced by GluN1-Ab.

The observed network reorganization with higher functional coupling and network hubness/clustering despite overall reduced functional connectivity seems antithetical, given: 1) the well-established disrupted LTP [6, 17, 95–99] and 2) previous reports of overall reduced excitatory synaptic strength onto CA1-PNs [18] (see also [6, 19, 20]). This apparent paradox, however, can be mechanistically reconciled as follows (Fig. 5). First, the pronounced coupling and hubness appear underpinned by the maintenance of relatively strong excitatory synapses, which likely form the backbone for these emerged network properties. Second, the concurrent overall reduced functional connectivity and the partitioning of network organization into a higher number of neuronal ensembles point towards an active synaptic weakening mechanism. We propose that the augmented NMDAR-dependent LTD preferentially targets and eliminates weaker functional connections, which could potentially be crucial for broader network integration and larger ensembles’ stability [34, 65–67]. Furthermore, this preferential filtering out of weaker synapses can make the background connectivity sparser, thereby increasing the relative prominence of functional network hubs and clustering. Of note, the abnormal LTD augmentation in CA1 was also reported in animal models of e.g. Huntington’s Disease, Fragile X Syndrome, and depression, all leading to hippocampal dysfunction [63, 64]. Collectively, we suggest that, besides the known pathophysiological changes (e.g. disrupted LTP, reduced AMPR/NMDAR currents), a competitive interplay between two concurrent synaptic phenomena (the preservation of effectively strong synapses and augmented LTD acting on weaker ones) may drive the abnormal functional reorganization observed, establishing a plausible substrate for the altered hippocampal dynamics in vivo. Analogous to the complex synaptic plasticity and homeostatic responses triggered by NMDAR antagonism (e.g., ketamine [100, 101]), chronic GluN1-Ab exposure likely induces active synaptic remodeling mechanisms that may contribute to sculpting the observed aberrant network topology. While characterizing the precise molecular cascades is beyond the current scope, our previous proteomic data [18] suggest that these chronic synaptic alterations likely involve regulation of protein translation and neurotrophic signaling pathways (e.g., BDNF/TrkB). Of note, we cannot exclude potential contribution of the altered intrinsic excitability of hippocampal PNs [18, 19] to these aberrant network patterns [33]. Moreover, these data may point to differential effect of GluN1-Ab on strength of functional connectivity at local neuronal microcircuits versus broader networks [22–24].

Importantly, aberrant functional rewiring and ensemble dynamics, similar to those we observed here, have been linked to the neuropsychiatric symptoms such as hallucinations, perceptual distortions, and cognitive impairment in schizophrenia [4, 37, 102, 103]. Analogous functional network pathologies are seen also in medically-induced loss of consciousness [104] and transgenic models of Alzheimer’s disease [81, 82, 105, 106]. Therefore, the network reorganization induced by GluN1-Ab may contribute to the disease phenotypes, including psychiatric symptoms e.g. psychotic experiences, derealization, and hallucination, as well as learning and memory deficits [1, 6, 7, 22]. Furthermore, the excessive similarity of internally-generated spatiotemporal activity patterns may contribute to the recently reported reductions in serial dependence (i.e. a readout of passive information maintenance across trials) in anti-NMDAR encephalitis patients [107] potentially through disturbing working memory traces [108, 109], and may also impair memory flexibility by reducing the repertoire of available activity patterns. Additionally, the pathological emergence of more numerous CA1 PN-ensembles and aberrant frequency and neuronal recruitment dynamics of SPW-Rs may also be detrimental to memory recall or ‘neural replays’ during memory consolidation [30, 35, 73, 74, 108, 110], given CA1’s crucial role in hippocampal-cortical memory circuits [111, 112]. Collectively, our identified organizational and dynamic alterations suggest a shift towards more stereotyped, potentially rigid neuronal communication, channeled via less frequent but potent synchronous events. While such aberrant structuring might strengthen the integration of specific, familiar signals within reinforced pathways [34, 109], this same rigidity, a feature also reported in CA1 activity patterns of other neurological disorders (e.g. dementia models) [90, 109, 113], can limit the network’s capacity for dynamic information processing [67, 90, 109, 113–115]. Such network flexibility is important for complex cognitive functions like encoding and retrieving diverse memories [108, 109, 116]. Crucially, however, our study primarily characterizes network alterations that likely underpin these higher-level processes, where future investigations, particularly in behaving animals, are needed to directly quantify their impact on network information processing and disease-relevant behaviors.

Despite providing valuable insights, the present study is also subject to some limitations. First, the choice of antibody used in the passive transfer encephalitis mouse model was determined by the high number and quality of previous studies using the same clone targeting the ATD of the NMDAR GluN1 subunit [9, 18–20, 117]. While using patient-derived, polyclonal CSF/serum might be more representative in reflecting diverse clinical conditions of NMDAR encephalitis patients, previous studies showed a high degree of consistency between the GluN1 clone used herein (003–102) and patient-derived polyclonal CSF regarding the major pathogenic functions, e.g. receptor internalization and NMDAR hypofunction [9, 10, 18]. Importantly, this particular Glun1-Ab clone (003–102) was tested in comparison to the patients CSF in a recent study, demonstrating similar pathogenic effects on CA1 network-activity ex vivo [18]. Moreover, using a monoclonal antibody in this study offers the advantage of reproducibility and consistency of antibody dosing.

An important limitation of the present study is the use of superficial isoflurane anesthesia, which modifies spontaneous activity (e.g., favoring slow oscillations [46–48]) compared to the awake state optimal for sensory processing studies. A specific concern, particularly in this disease model, is the possibility that GluN1-Ab could alter anesthetic sensitivity, potentially leading to a differential network state between groups despite identical anesthetic delivery. However, our simultaneous LFP recordings from the CA1 *stratum radiatum* argue against this possibility. Most critically, we found no significant difference between Ctrl-Ab and GluN1-Ab groups in the LFP slow-wave power spectra which are highly sensitive to anesthetic state [45–48, 118, 119]. Furthermore, the LFP from both groups was dominated by delta-band oscillations, consistent with our goal of investigating intrinsic, sensory-minimized [45, 46] network dynamics [25–28]. Additionally, we acknowledge that our reported properties of SPW-Rs are restricted to LFP recordings acquired by single-tip electrodes, as high-density laminar recordings are required for a full characterization [73, 75, 76]. Nevertheless, we emphasize that these network results should not be directly extrapolated to predict network function during active, sensory-driven cognitive tasks in the awake condition. Instead, they suggest how the potential neural substrate of hippocampal network organization and spontaneous activity are pathologically altered by GluN1-Ab. Future studies in awake, behaving animals will therefore be essential to determine how these profound alterations in the intrinsic network substrate ultimately impact cognitive processing and behavior.

Finally, the complex network pathology identified here–reduced overall functional connectivity contrasting with pronounced functional coupling, clustering, and hubness–highlights potential targets for therapeutic intervention. Augmenting signaling through remaining functional NMDARs using Positive Allosteric Modulators (NMDAR-PAMs) represents one promising approach, as these agents reverse disease symptoms and key pathogenic events in active and passive disease mouse models [97, 120]. Such interventions might counteract the augmented LTD and potentially rebalance the aberrant network organization and dynamics within hippocampal microcircuits. Furthermore, previous results in hippocampal slices have shown the efficacy of AMPAR-PAMs in reducing network hypersynchrony ex vivo through normalizing gamma oscillations power [18]; a phenomenon related to the intrinsic hypersynchrony-prone network state identified in our in vivo model. Thus, the detailed characterization of antibody-induced network abnormalities shown here offers a valuable and disease-relevant in vivo platform for assessing the potential of targeted pharmacological interventions to restore physiological network organization and dynamics.

Together, our findings reveal a profound functional rewiring of the intrinsic building blocks of hippocampal circuitry which not only leads to an intrinsic network state favoring hypersynchrony but also alters its intrinsic organization and spatiotemporal dynamics, thus providing new mechanistic insights into the consequences of NMDAR hypofunction and the pathomechanisms of anti-NMDAR encephalitis.

## Materials and methods

A detailed description of our experimental methods, as well as all analysis and simulations used in this study, is provided in the Supplementary Information (Supplementary Methods).

## Supplementary information

Supplementary Information

Supplementary Table 1

## Data Availability

Statistical data associated with this study are presented in Supplementary Table 1. Any additional information required to reanalyze the data reported in this work paper is available from the corresponding authors upon request.

## Code availability

This paper does not report original code central to the main results. MATLAB toolboxes for the Brain Connectivity Toolbox (brain-connectivity-toolbox.net), NoRMCorre (https://github.com/porteralab/NoRMCorre), *gini* function (https://www.mathworks.com/matlabcentral/fileexchange/28080-gini-coefficient-and-the-lorentz-curve), UFARSA (https://github.com/VahidRahmati/UFARSA), FluoroSNNAP (https://www.seas.upenn.edu/~molneuro/software.html), FRACI (https://github.com/CABSEL/FARCI), as well as the MESc (https://femtonics.eu/) and Fiji (https://fiji.sc/) software programs are publicly available online.
