# Supplementary material for: Anti-NMDAR encephalitis impairs intrinsic hippocampal dynamics through neuronal hypercoupling, hub dominance, and aberrant ensembles
Source: Mol Psychiatry. 2026 Mar 31;31(8):4550–62. doi: 10.1038/s41380-026-03568-6 (PMC13364672; doi:10.1038/s41380-026-03568-6)
Supplement: Supplementary file 1 — Supplementary Information [file 41380_2026_3568_MOESM1_ESM.pdf]

Supplementary Information

Anti-NMDAR encephalitis impairs intrinsic hippocampal dynamics through neuronal hypercoupling, hub dominance, and aberrant ensembles

Vahid Rahmati, Jürgen Graf, Mihai Ceanga, Dario Cuevas Rivera, Holger Haselmann, Sabine Liebscher, Harald Prüss, Knut Holthoff, Knut Kirmse, Christian Geis

Corresponding authors:

Vahid Rahmati: Vahid.Rahmati@med.uni-jena.de

Christian Geis: Christian.Geis@med.uni-jena.de

Supplementary Figures

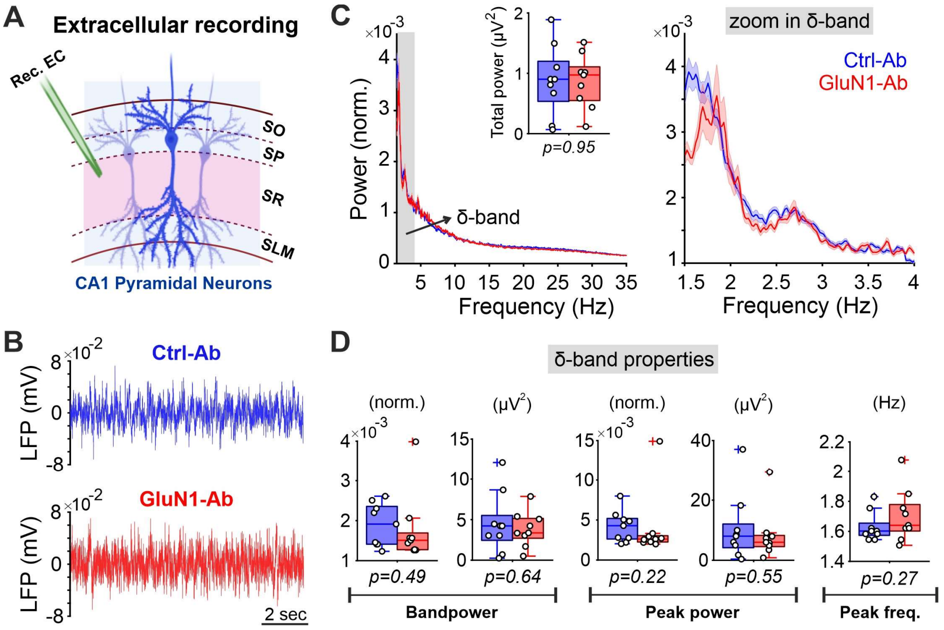

Supplementary Figure 1. LFP power spectra are comparable between Control-Ab and GluN1-Ab groups, suggesting a similar anesthetic state. (**A**) Schematic of *in vivo* local field potential (LFP) recordings from the proximal *stratum radiatum* (SR) of hippocampal CA1, performed simultaneously with two-photon imaging of the *stratum pyramidale* (SP; Fig. 1A). Created in BioRender; Geis, C. https://BioRender.com/9qs1fwa (2026). (**B**) Representative 10-second LFP traces from one-hour recordings for each group. (**C**) LFP power spectra do not differ between groups. *Left*: Power spectrum normalized to total power (1.5–100 Hz) per mouse to compare spectral shape. The δ-band (1.5–4 Hz), containing the dominant oscillatory activity, is highlighted. Inset shows absolute total power per mouse is also not different between groups. *Right*: Zoomed-in view of the δ-band, showing a peak consistent with slow-wave activity observed during non-REM sleep and under light anesthesia. (**D**) Key δ-band spectral properties are unaltered between groups. Boxplots compare both absolute (μV^2) and normalized band power, peak power, and peak frequency within the δ-band for each mouse. The lack of significant differences in these features indicates a comparable net synaptic activity state within the *stratum radiatum* across Ctrl-Ab and GluN1-Ab groups.

Curves represent mean ± SEM across mice. Boxplots show median and interquartile range; dots represent individual mice. Sample sizes: (C, D) n=9 mice/group. Statistical comparisons: two-sample t-tests (C, D [bandpower μV^2]) or Mann-Whitney U tests (D [norm. bandpower, peak power, preak frequency]); see Supplementary Table 1.

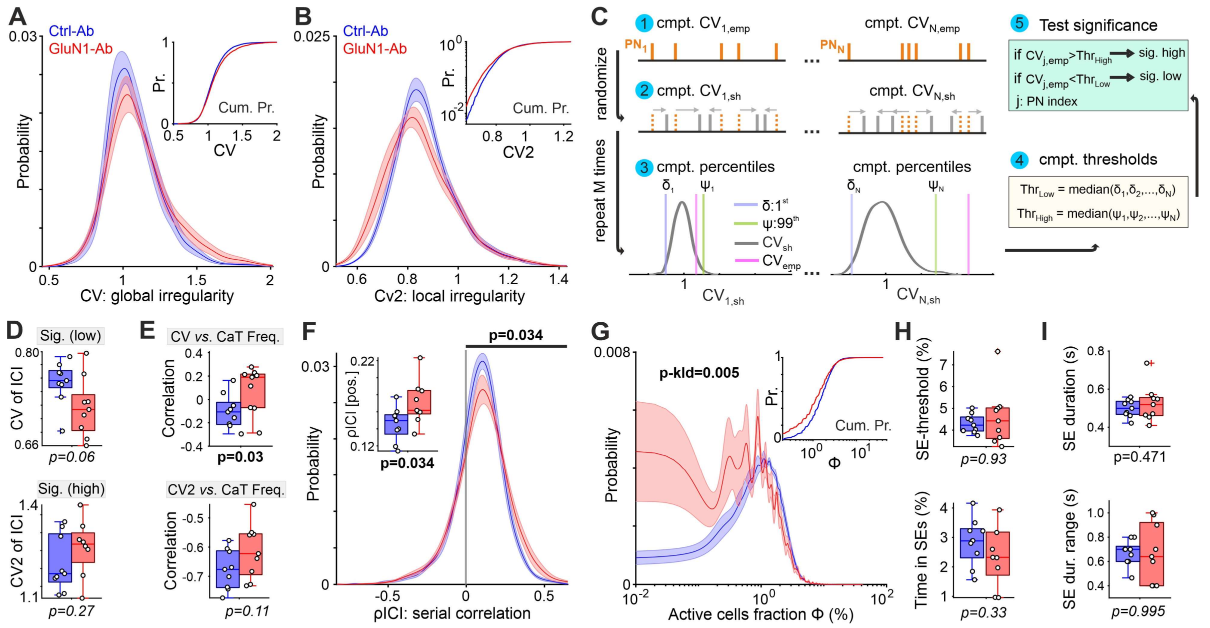

Supplementary Figure 2. Complementary results related to Figure 1. (**A**,**B**) Distributions of global (A) and local irregularity of putative neurons' (PNs') ICIs (B). Same format as in (Fig. 1E). (**C**) Schematic illustrating the procedure for statistical assessment of a PN's CaT irregularity (CV or CV2), using CV as an example. (1) Calculate the PN's empirical CV (CV_{emp}). (2) Randomly shuffle the CaT event times for this specific PN and re-calculate the CV (CV_{sh}). Note that this shuffling preserves the neuron's event count while disrupting the precise timing of its events. (3) Repeat step (2) for 500 times, yielding a neuron-specific distribution of 500 CV_{sh} values. Determine the 1th (δ) and 99th (Ψ) percentiles of this distribution for this neuron. (4) Repeat steps 1–3 for all N neurons. Derive two population-level thresholds: Thr_{Low} = median(δ_1,δ_2,...δ_N) and Thr_{High} = median(Ψ_1, Ψ_2, ,... Ψ_N). (5) Compare the empirical CV_{emp} of each PN to these thresholds (Thr_{Low} and Thr_{High}) to assess whether it is significantly low or high relative to the population baseline (See Supplementary Methods for more detail). (**D**) The irregularity level of PNs with significantly low CV and high CV2 values. (**E**) The relationship between ICIs' global irregularity (CV) and CaT frequency of PNs (top), and between ICIs' local irregularity (CV2) and CaT frequency of PNs (bottom). (**F**) Redistribution of serial correlation of PNs' ICIs (ρICI) towards higher values under GluN1-Ab, reflecting reduced PNs' local firing irregularity. Inset: boxplots of mean of positive ρICI per mouse. (**G**) Redistribution of network activity towards lower values. Same format as in Fig. 1E. (**H**) SE-detection threshold (top) and the fraction of the summed duration of all SEs relative to the entire recording time (bottom). (**I**) The mean duration of SEs (top) and the range of SE durations defined as the 95th *minus* 5th percentile of all SE durations per mouse (bottom).

Curves represent mean ± SEM across mice. Boxplots show median and interquartile range; dots represent individual mice. Sample sizes: (A, B, D–I) n=9 mice/group (total cells: 4279 Ctrl-Ab, 3200 GluN1-Ab). Statistical comparisons: two-sample t-tests (A, B, D–F, H [bottom], I), Mann-Whitney U tests (H [top]), or permutation tests (G); see Supplementary Table 1.

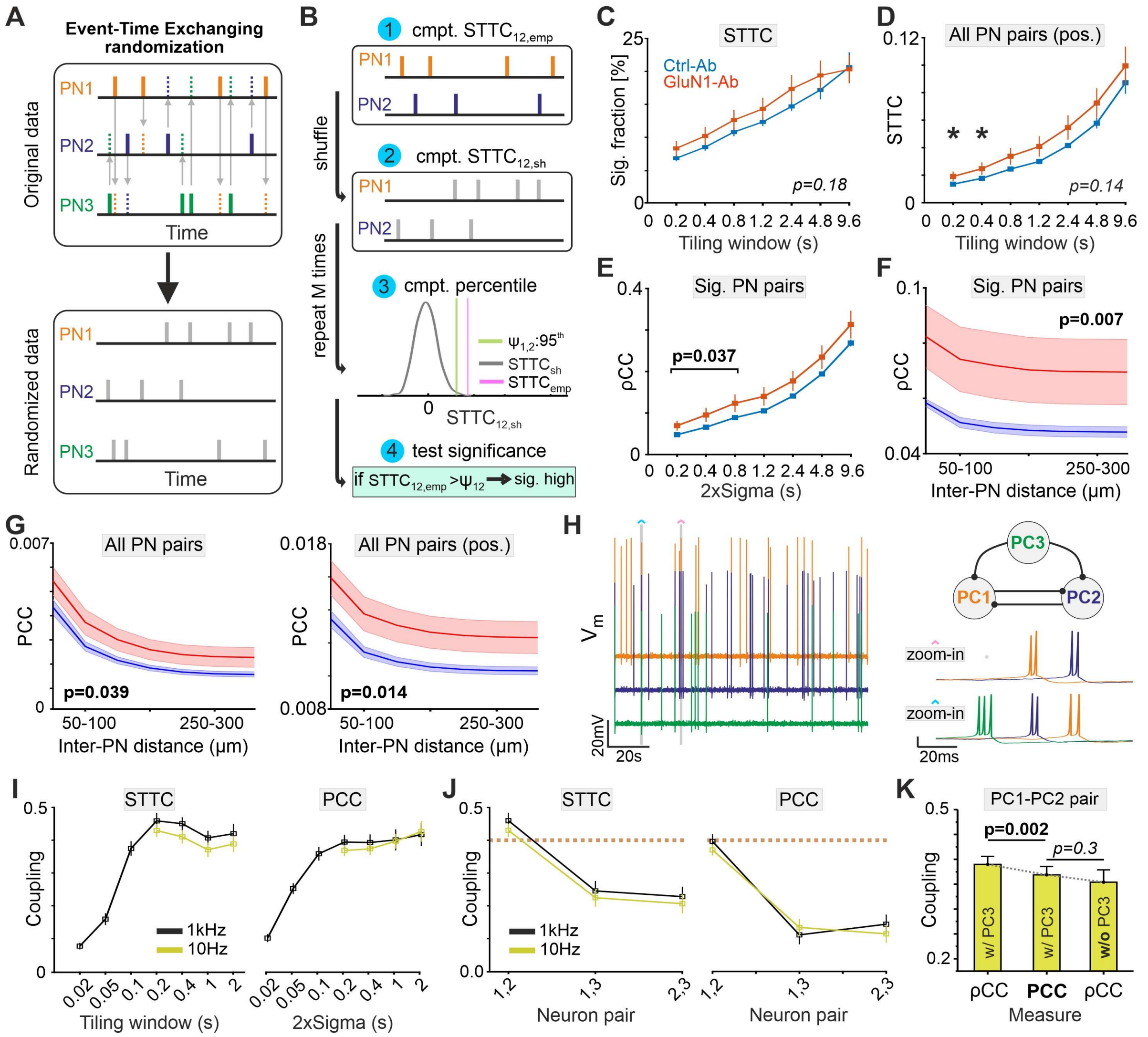

Supplementary Figure 3. Complementary results related to Figure 2. (**A**) Schematic of Event-Time Exchanging (ETE) randomization used to generate surrogate data. Event times from all neurons are pooled and randomly reassigned, preserving individual neuron event counts while disrupting precise inter-neuron temporal relationships. (**B**) Schematic illustrating the procedure to evaluate the statistical assessment of neuronal coupling measures, using STTC between a putative neuron (PN) pair as an example. (1) Calculate the empirical STTC for each PN pair using their original event trains ($STTC_{emp}$). (2) Perform one ETE shuffle across the entire event times of the population (as shown in [A]). Using the resulting surrogate event trains, re-calculate the STTC for each PN pair ($STTC_{sh}$). (3) Repeat step (2) M times (e.g. 500 times) to generate a pair-specific distribution of M $STTC_{sh}$ values for each PN pair. Determine the significance percentile (e.g. 95th percentile, Ψ) of this shuffle-based distribution for each pair. (4) Compare the empirical $STTC_{emp}$ of each PN pair to its own significance threshold (Ψ) to assess whether it is significantly high (i.e. whether $STTC_{emp} > \Psi$; see Supplementary Methods). (**C**) Fraction of PN pairs with significantly high STTC, quantified at various timescales. Same format as in Fig. 2F. (**D**) Effect of GluN1-Ab on STTC of all PN pairs with positive value, at various timescales. Same format as in Fig. 2F. (**E**) Neuronal coupling based on Pearson correlation coefficient of Gaussian-smoothed event trains (ρCC), computed at various timescales. Same format as in Fig. 2F. Pairs with significantly high ρCC (using the same approach as in [B]) were considered. (**F**) Relationship between PN pairs with significant ρCC and their Euclidean somatic distance. Same format as in Fig. 2E. (**G**) Relationship between PCC and Euclidean somatic distance of PNs. Same format as in Fig. 2E. (**H**) Setup for simulating a neuronal network of three pyramidal cells (PCs), each amenable of firing bursts. *Left:* Example

membrane potential traces (V_m) simulated for 120 seconds. *Right:* Schematic of the network: PC1 and PC2 are recurrently connected, and PC3 projects unidirectionally to both PC1 and PC2. Each neuron also received independent random background input. Simulations (n=10 trials with different random seeds) were run at 0.05 ms resolution; action potentials were detected and then downsampled, using binary binning, to 1 kHz (1 ms resolution) and 10 Hz (100 ms resolution, mimicking our experimental data). Zoom-in panels show examples of bursting activity, and correlated firing potentially induced between PC1 and PC2 by the activity of PC3 (bottom). (**I**) Robustness of STTC and PCC calculations to lower temporal resolution at relevant analysis timescales. Trajectories show STTC (left) and PCC (right) for the PC1-PC2 pair, calculated across various analysis timescales (tiling window or 2xSigma). Each plot compares the coupling values obtained from high-resolution (1kHz, black) and low-resolution (10Hz, green) versions of the simulated spike trains. Note the similarity in trajectory shapes for each measure between the two resolutions for analysis timescales longer than 100 ms. This indicates that both STTC and PCC capture similar coupling dynamics at these longer timescales despite the lower data resolution, validating their use with the ~100ms-resolution experimental data. Timescales shorter than 100 ms are not applicable for 10 Hz data. Similar robustness was observed for ρCC (not shown). (**J**) Preservation of relative coupling strengths between different neuron pairs at low temporal resolution. Coupling strength (STTC and PCC) calculated at a fixed timescale (0.2 s) for different pairs (PC1-PC2, PC1-PC3, PC2-PC3) at both 1 kHz and 10 Hz resolution. Note that the higher coupling strength of the recurrently connected PC1-PC2 pair compared to pairs involving PC3 is maintained at the lower resolution. (**K**) Partial correlation coefficient (PCC) reduces the indirect influence of other network neurons to estimate the direct coupling of a neuron pair, even at a low temporal resolution. Comparison of PCC and standard Pearson Correlation (ρCC) for the PC1-PC2 pair calculated at 10 Hz resolution. ρCC is technically similar to PCC but can be inflated if both PC1 and PC2 are influenced by a third neuron, such as PC3 in this simulation (activity potentially correlating PC1 and PC2 is visible in bottom zoom-in of H). In contrast, PCC attempts to control for such indirect influences and isolate the direct relationship. The ρCC calculated after computationally removing PN3 ("w/o PC3") represents the target 'true' direct correlation. Note that the PCC value (middle bar) is lower than the standard ρCC (left bar) and closer to the ρCC that reflect the direct connection (right bar; "w/o PC3"). This demonstrates PCC's ability to partially correct for the confounding influence of PC3 and provide a more accurate estimate of the direct interaction, even when calculated using data with 10 Hz (100 ms) temporal resolution.

Curves represent mean ± SEM. Boxplots show median and interquartile range; dots represent individual mice or simulation trials. Sample sizes: (C–G) n=9 mice/group (total cells: 4279 Ctrl-Ab, 3200 GluN1-Ab); (I–K) n=10 simulation trials. Statistical comparisons: paired sample t-tests (K), Mann-Whitney U tests (asterisks in D), or permutation tests (C–G); see Supplementary Table 1.

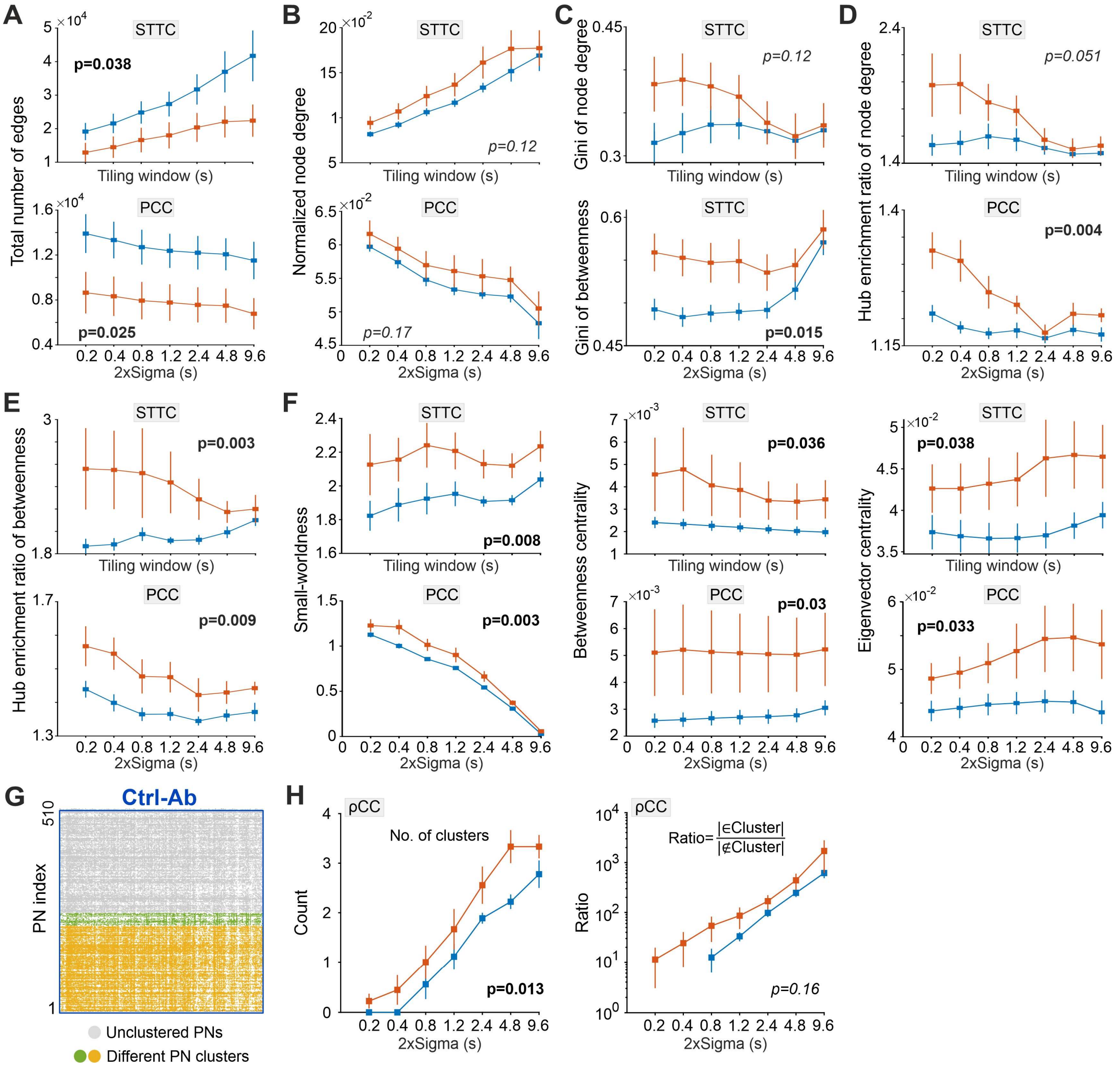

Supplementary Figure 4. Complementary results related to Figure 3. (**A**) Reduction in total number of graph edges (functional connections within network) under GluN1-Ab. Same format as in Fig. 2F,G. (**B**) Normalized node degree, adjusting for network size, remains unchanged under GluN1-Ab. The degrees were computed based on STTC (top) or PCC (bottom) indices. (**C**) Gini coefficient of betweenness (top) and node degree (node degree) based on STTC coupling results. The Gini coefficient quantifies the distribution inequality in terms of its shape and thus is normalization-independent. (**D,E**) Increased hub enrichment ratio of node degree (D) and betweenness (E) under GluN1-Ab. This metric computes the prominence of upper-tail of a distribution using its percentiles (the ratio of 75th and 50th percentiles), and is thus normalization-independent. (**F**) Increased small-worldness (measuring the balance between local clustering and global connectivity in a network), betweenness centrality (measuring the extent to which a putative neuron [PN] acts as a go-between for other PNs), and eigenvector centrality (measuring the importance of a node in a network based on its connections to other highly central nodes) under GluN1-Ab. (**G**) Rastergram of the FOV shown in Fig. 3G under Ctrl-Ab. Same format as in Fig. 3H. (**H**) Using ρCC, instead of STTC (Fig. 3I), led to similar ensemble results with an increased number of detected PN ensembles (left) and an unchanged ratio of the PNs in ensembles to those unaffiliated PNs (right), per FOV.

Curves represent mean ± SEM across mice. Boxplots show median and interquartile range; dots represent individual mice. Sample sizes: (A–F, H) n=9 mice/group (total cells: 4279 Ctrl-Ab, 3200 GluN1-Ab). Statistical comparisons: permutation tests (A–F, H); see Supplementary Table 1.

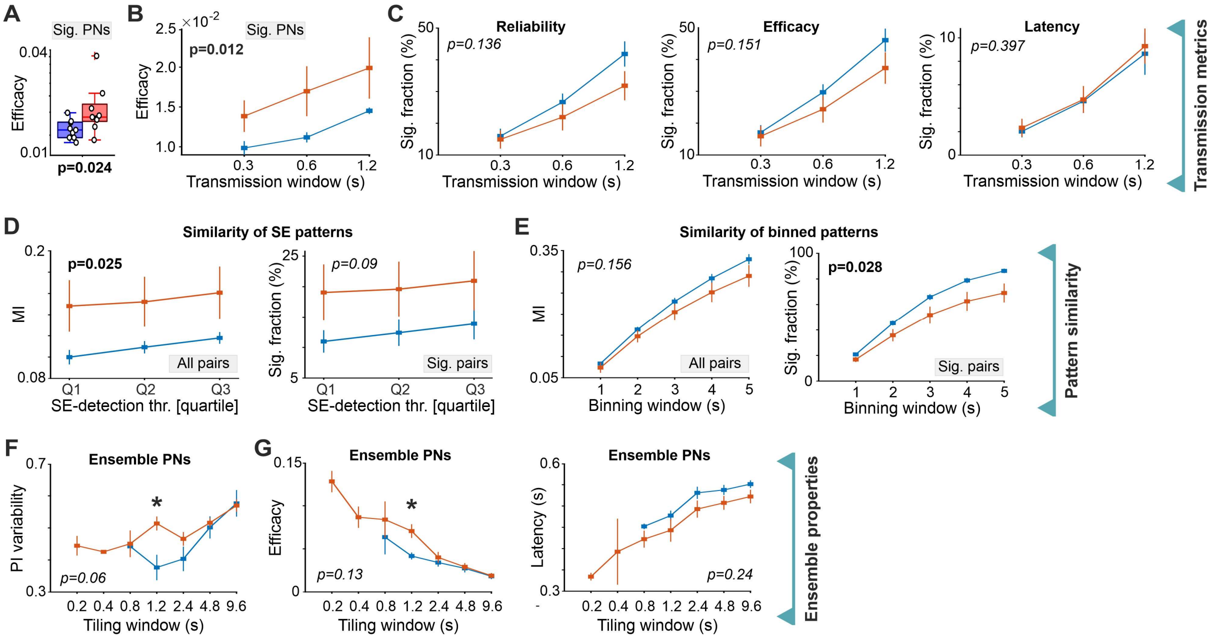

Supplementary Figure 5. Complementary results related to Figure 4. (**A**) Increased efficacy of individual putative neurons' (PNs') transmission to others (sig. PNs). Transmission window was set to 0.6 s (see also Fig. 4B). (**B**) Same as in Fig. 4C, but for the transmission efficacy (sig. PNs). (**C**) Fraction of PNs with significant transmission reliability (left), efficacy (middle), and latency (right). (**D**) Left: excessive similarity between SE binary patterns (all pairs) under GluN1-Ab. Right: Unchanged fraction of SE binary pattern pairs with significantly high MI under GluN1-Ab. Same format as in Fig. 4F bottom. (**E**) Similarity of spatial binary patterns as a function of bin-size used for binning the recording time to extract the patterns. Left: matching index (MI) for all pairs of binned patterns. Right: fraction of the significant pairs. Note the decreasing trend in the fraction of significant pairs at longer timescales (right). (**F**) Variability of participation index (PI) of PNs to their respective ensembles (see Fig. 3G–I), at various timescales. Same format as in Fig. 2F. Variability was computed using coefficient of variation of PIs. (**G**) Same as (F), but for the efficacy (left) and latency (right) of ensemble PNs' transmission to other PNs. Transmission window was set to 1.2 s.

Curves represent mean ± SEM across mice. Boxplots show median and interquartile range; dots represent individual mice. Sample sizes: (A–G) n=9 mice/group (total cells: 4279 Ctrl-Ab, 3200 GluN1-Ab). Statistical comparisons: two-sample t-tests (asterisk in G), Mann-Whitney U tests (A, asterisk in F), or permutation tests (B–G); see Supplementary Table 1.

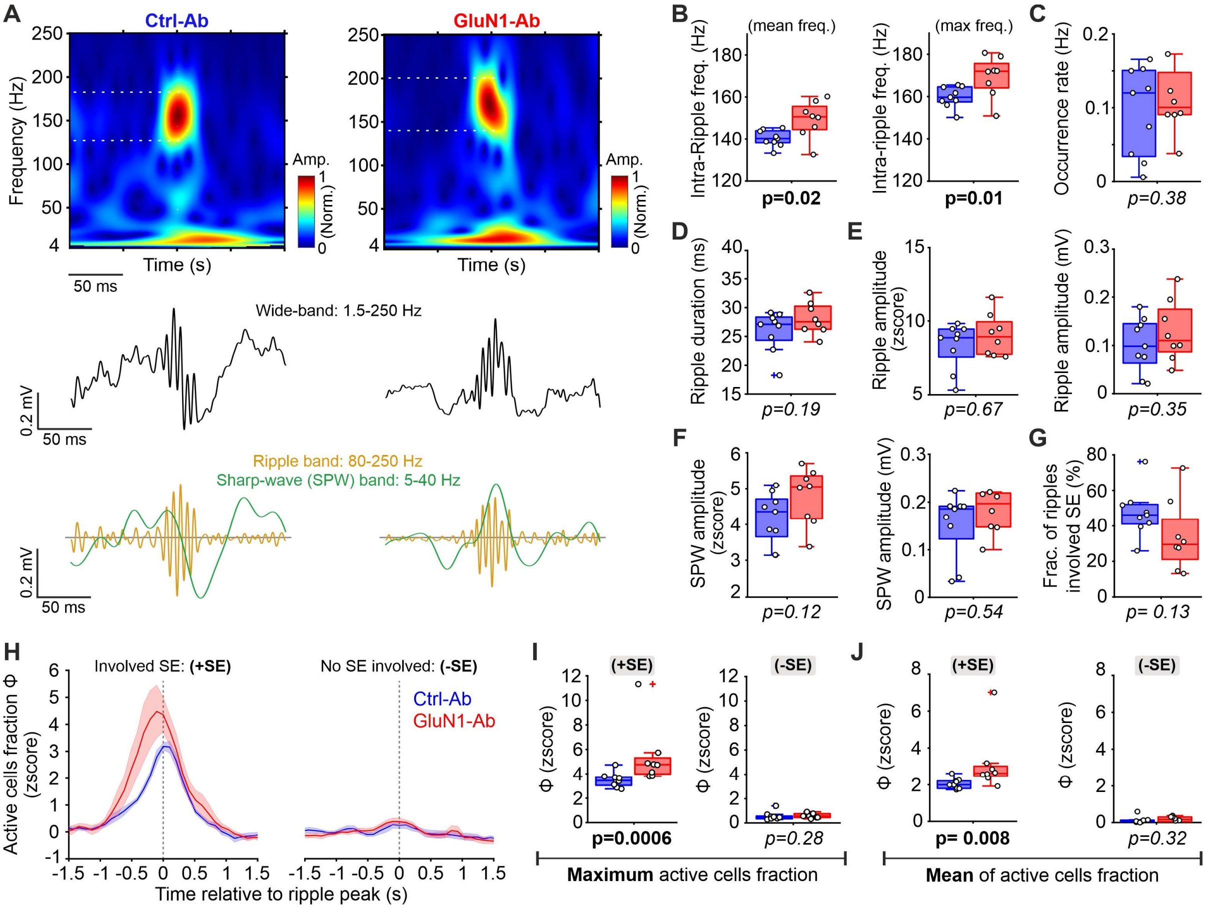

Supplementary Figure 6. GluN1-Ab induces faster ripples and amplifies SPW-R-associated synchronous events. (**A**) Top: Morlet wavelet time-frequency spectrograms of representative single sharp-wave ripple (SPW-R) events from LFPs recorded *in vivo* simultaneously with two-photon imaging for about 1 hour (see also Supplementary Fig. 1). A shift toward higher intra-ripple frequencies is visible in the GluN1-Ab example compared to Control-Ab. Bottom: Corresponding traces of the SPW-R complex shown above. Black traces show the wide-band LFP (1.5–250 Hz). Colored traces show the corresponding filtered signals for the ripple-band (orange; 80–250 Hz) and sharp-wave band (green; 5–40 Hz); gray line represent 0 mV. Note that while the recordings targeted the proximal *stratum radiatum*, sharp-wave deflections can appear negative or positive depending on the precise electrode depth *in vivo* relative to the CA1 dipole (*stratum radiatum* vs. *pyramidale*); detection was therefore performed using absolute amplitude. (**B**) Quantification of intra-ripple frequency. The mean (left) and maximum (right) instantaneous frequencies were calculated for each SPW-R event and averaged per mouse. GluN1-Ab mice exhibit faster average oscillations and reach higher peak frequencies during ripples. (**C**) The occurrence rate of SPW-Rs is unchanged between groups. (**D**) The duration of ripple events is preserved. (**E**) Peak amplitude of the ripple oscillation envelope, shown as z-score (left) and absolute voltage (right), is unchanged. (**F**) Peak amplitude of the sharp-wave deflection, shown as z-score (left) and absolute voltage (right), is unchanged. (**G**) The fraction of SPW-Rs coinciding with a Synchronous Event (SE) within a ±300 ms window around the ripple peak. There was no significant difference in the coincidence rate between groups. (**H**) Peri-Event Time Histograms (PETH) of the fraction of active cells Φ(t) (z-scored to baseline: -3.5 to -1.5 s) centered on the ripple peak. Left: PETH for the subset of SPW-Rs coincident with an SE [(+SE)]. Note the sharp rise in population activity, which is amplified in GluN1-Ab mice. Right: PETH for SPW-Rs not coincident with an SE [(-SE)], showing no distinct population activation in both groups. (**I**) Maximum z-scored population activity during (+SE) and (-SE) events. (**J**) Mean z-scored population activity during (+SE) and (-SE) events (averaged over ±0.5 s). Note the amplified network synchrony during coincident events in the GluN1-Ab group.

Boxplots show median and interquartile range; dots represent individual mice. Sample sizes: (B–J) n=9 (Ctrl-Ab) / 8 (GluN1-Ab) mice. Statistical comparisons: two-sample t-tests (C–D, E [right], F [left], G), or Mann-Whitney U tests (B, F, E [left], F [right], I–J); see Supplementary Table 1.

Supplementary Methods

Materials availability

This study did not generate new reagents.

Experimental model

- **Animal model of NMDAR-encephalitis**

Thirty three male C57BL/6J mice were housed under standard conditions at a controlled temperature (21 ± 1°C) and humidity (55 ± 10%) with 12-h illumination cycles, and food and water available *ad libitum*. Animal experiments were performed in accordance with the ARRIVE guidelines for reporting animal research and the experimental protocol was in accordance with European regulations (Directive 2010/63/EU) and was approved by the local animal welfare committee (Thüringer Landesamt für Lebensmittelsicherheit und Verbraucherschutz, 02–059/13 and UKJ-23-002), similarly to [1]. Sixteen-weeks old (25–30g) mice were implanted with biventricular osmotic pumps (model 1002, Alzet, Cupertino, CA) with the following characteristics: volume 100 µL, flow rate 0.25 µL/h, and duration 14 days, as previously reported [1,2]. The day before surgery the two pumps were each filled with 100µL of 1µg/µL human monoclonal NMDAR-Ab (#003–102) or Control-Ab (#mGO53) [3]. NMDAR-Ab #003–102 targets the lower lobe of the NMDAR GluN1 ATD and was shown to reproduce similar molecular pathology as polyclonal human CSF IgG, e.g. receptor crosslinking and internalization leading to reduction in NMDAR-mediated currents and impairing hippocampal LTP [4–9]. Control-Ab #mGO53 is a nonreactive, isotype-matched human IgG1 antibody [10], widely used and well validated negative control antibody employed in autoimmune encephalitis research[1,3,11–13]. Both antibodies were produced by H. Prüss lab[3]. Mice under isoflurane anesthesia were placed in a stereotactic frame, and a bilateral cannula (model 3280PD2.0/SP, PlasticsOne) was inserted into the ventricles (coordinates: 0.2 mm posterior and ±1.00 mm lateral from bregma, depth 2.2 mm). The cannulas were connected to two subcutaneously implanted osmotic pumps on the back of the mice.

Method details

- **Surgical preparation for *in vivo* imaging**

Thirty minutes before starting the preparation, 200 mg/kg metamizol (Novacen) was administered subcutaneously for analgesia. The animals were then placed on a warm platform and anesthetized with isoflurane (3.5% for induction, 1–2% for maintenance) in pure oxygen (flow rate: 1 l/min). A drop of eye ointment (Vitamycin) was applied to lubricate their eyes. The intraventricular cannula and osmotic pumps were carefully removed. For local analgesia, the skin was infiltrated with 2% lidocaine (s.c.). The scalp and periosteum were removed, and a custom-made plastic chamber with a central borehole (Ø 4 mm) was affixed to the skull using cyanoacrylate glue (UHU) (2.5 mm posterior from bregma and 2.2 mm lateral from midline). For the hippocampal window preparation[14], the plastic chamber was securely attached to a preparation stage and superfused with warm artificial cerebrospinal fluid (aCSF) containing (in mM): 125 NaCl, 4 KCl, 25 NaHCO3, 1.25 NaH2PO4, 2 CaCl2, 1 MgCl2 and 10 glucose (pH 7.4, 35–36°C). A circular hole was drilled into the skull using a tissue punch (Ø 2.7 mm). The underlying cortical tissue and parts of corpus callosum were carefully removed by aspiration using a vacuum supply and a blunt 30G needle, taking care not to damage the alveus fibers. Once bleeding stopped, the animal was transferred to the microscope stage. During *in vivo* recordings, body temperature was continuously monitored and maintained

close to physiological values (36–37°C) by means of a heating pad and a temperature sensor placed beneath the animal. Spontaneous respiration was monitored using a differential pressure amplifier (Spirometer Pod and PowerLab 4/35, ADInstruments). Shortly after transfer, Isoflurane was reduced to 0.6% (flow rate: 1 l/min). Recording of spontaneous activity commenced 60 min afterwards. At the end of each experiment, the animal was decapitated under deep isoflurane anesthesia.

- **Two-photon Ca^{2+} imaging *in vivo* and LFP recording**

The recording chamber was continuously superfused with aCSF (as above). *Stratum pyramidale* in the CA1 region was loaded with the membrane-permeable Ca^{2+} indicator Oregon Green 488 BAPTA-1 AM (OGB-1, 500 µM, pipette resistance: ~3 MΩ, pressure: 7 PSI, injection time: 1.5 min) using the multi-cell bolus-loading technique[15]. For local field potential (LFP) recordings, a tungsten microelectrode (Tunglass-1, 0.8 MΩ impedance, Kation Scientific) was positioned just above the hippocampal formation. After the aCSF was removed, the hippocampal window was filled with agar (1.5%, in 0.9 mM NaCl) and covered with a custom-made cover glass. Once the agar solidified, the chamber was reperfused with ACSF. The microelectrode was then slowly lowered into the CA1. Upon detecting pronounced multi-unit activity (MUA) in the *stratum pyramidale* (SP), the electrode was further advanced to target the proximal *stratum radiatum* (SR), approximately 250-300 µm below the hippocampal surface. To prevent photoelectric artifacts in the imaging data caused by the laser scanning, the LFP microelectrode was positioned approximately 1-2 mm laterally from the edge of the imaging field of view (FOV). To allow for de-esterification, recordings commenced approximately 60 minutes after OGB-1 injection. Imaging was performed using an acousto-optic deflection (AOD) two-photon laser-scanning microscope controlled by the MES software (Femto3D ATLAS, Femtonics). Fluorescence excitation at 800 nm was provided by a tunable Ti:Sapphire laser (Chameleon Ultra II, Coherent) through a 20×/1.0 NA water immersion objective (XLUMPLFLN 20XW, Olympus). Emission light was separated from excitation light with a primary dichroic mirror (700 nm) and an IR blocker (700 nm) and finally detected by photomultiplier tubes (16 bit, H11706P-40, Hamamatsu). The high-speed arbitrary frame scanning mode of ATLAS enabled a fast frame rate of 38.81 Hz. The FOV was 329×329 µm at a pixel resolution of 0.9 µm/pixel (366×366 pixels, dwell time: ~0.14 ns). Spontaneous activity in the dorsal CA1 was recorded for a total duration of 60 min (57.88 ± 0.91 min, n=18 mice; mean ± SEM). A single FOV was recorded per mouse. Data were acquired using MESc (Femtonics). LFP signals were acquired simultaneously to two-photon imaging using an npi ELC-03XS amplifier (npi electronic GmbH), with a hardware highpass filter of 0.5 Hz and lowpass filter of 3 kHz. Amplified signals were then digitized at 20 kHz using a 16-bit AD/DA board (PowerLab 4/35, ADInstruments) and recorded using LabChart 8 software (ADInstruments).

- **Analysis of imaging data**

For each FOV, image stacks were registered using NoRMCorre [16]. For residual drift detection, a supporting metric was calculated as the Pearson correlation coefficient between the template image used for stack registration and the images of the registered image stack. Time periods with residual drift (e.g. by abrupt mouse movement) and distorted frames (e.g. by mechanical artifacts) were then visually identified (by inspecting the supporting metric and the registered image stack) and considered as missing values in subsequent analyses [17]. This step yielded to an available recording time of about 51.73 ± 1.28 min (n=18 mice; mean ±

SEM) per FOV. We also removed 10 pixels from each end of FOV to avoid potential instability in alignment at the borders. For cell segmentation, we first smoothed the registered stack using a 3D Gaussian smoothing kernel (*imgaussfilt*3 Matlab function) of size [2 pixels, 2 pixels, 3 frames], followed by adapting the widely-used independent component analysis. The detected raw ROIs were then visually double-checked with the registered image stack. We then down-sampled the (non-smoothed) registered data to 9.7 Hz (~100 ms)[17] to improve the signal-to-noise ratio of traces. To isolate neuronal signals, we applied strict exclusion criteria to separate neurons from glial cells (e.g., astrocytes), which are also labeled by OGB-1. Glial cells were identified and excluded based on distinct morphological and functional properties[18]: 1) smaller, irregular somata compared to the larger, rounder neurons; 2) significantly higher baseline brightness; and 3) much slower calcium transient dynamics (rise/decay times > several seconds) as compared to those of neurons.
The final retained ROIs were considered as the somata of putative neurons (PNs). While the *stratum pyramidale* mainly comprises pyramidal cells (>90%), we acknowledge that a small fraction of the detected somata might correspond to interneurons[19,20]. We detected an average of 475.4 ± 32.6 cells per FOV in the Ctrl-Ab group (n=9 mice; range: 299–629 cells; total: 4279 cells) and 355.6 ± 45.9 cells per FOV in the GluN1-Ab group (n=9 mice; range: 105–544 cells; total: 3200 cells). The individual cell counts for each mouse in the Ctrl-Ab group were: 522, 453, 473, 507, 299, 500, 629, 538, 358; and for the GluN1-Ab group: 426, 339, 440, 105, 544, 276, 355, 226, 489. For each ROI, we obtained the mean F(t) by frame-wise averaging across all pixels of that ROI in registered stack. The somatic fluorescence traces were obtained as relative changes from resting fluorescence levels ($\Delta F/F_0$). The resting fluorescence $F_0(t)$ was defined as the moving median over ~30 seconds. For overlapping ROIs, we extracted the fluorescence trace of each ROI based on its non-overlapping pixels. For each ROI, CaT onsets, as a proxy for firing activity, were extracted from its $\Delta F/F_0$ trace using UFARSA, a general-purpose event detection routine [21]. We used default parameter values, with a detection (leading) threshold of 2 SD_{noise}, providing a reliable balance between sensitivity (detecting real transients) and specificity (rejecting noise fluctuations); SD_{noise} is the estimated standard deviation of the signal noise (for more details, see [21]). Reconstructed CaT onsets were translated into a binary activity vector (1 – event, 0 – no event) and used for the following analyses. In total, 10 FOVs were recorded for each group (1 FOV per mouse), and one FOV from each group was excluded due to the instability of recordings (e.g. excessive motion artifacts or poor signal quality), according to predefined quality control criteria.

- **Analysis of LFP signals**

For each mouse, an extracellular LFP signal was recorded simultaneously with two-photon imaging for approximately 1 hour at a sampling frequency of 20 kHz. Preprocessing of the raw LFP signal was performed offline using custom-written code in Matlab. First, periods containing potential movement artifacts were identified by visual inspection and excluded. The signal was then digitally band-pass filtered in the range of 1.5 Hz and 251 Hz using a Kaiser window finite impulse response (FIR) filter with a 1 Hz transition bandwidth, 60 dB stopband attenuation, and 1% passband ripple. This digital high-pass cutoff at 1.5 Hz was chosen to create a sharp, well-defined frequency bound, removing both slow signal drift and residual low-frequency noise below our primary band of interest that may not have been fully attenuated by the amplifier's 0.5 Hz hardware filter. Power line interference at 50 Hz and its harmonics were removed using a notch filter (implemented with the *iirnotch* and *filtfilt* functions). Following filtering, the signal was downsampled to 5 kHz to reduce computational

load for subsequent analyses. Power spectral density (PSD) of the signal was calculated using Welch's method (*pwelch* function) with 30-second Hamming windows and 50% overlap between segments. For specific frequency bands of interest (e.g., the δ-band, 1.5–4 Hz), the bandpower was calculated by averaging the power values across all frequency bins within that band's limits. For normalization, the power at each frequency bin or the bandpower was divided by the total power of the signal (1.5–100 Hz).

To investigate the properties of sharp-wave ripples (SPW-Rs), we further filtered the downsampled signal into two distinct frequency bands using the abovementioned zero-lag FIR filters: a ripple band (80–250 Hz) and a sharp-wave band (5–40 Hz)[22,23]. The 80–250 Hz band was chosen, in line with recent literature, to robustly capture ripple events under anesthesia, which can occur at lower frequencies than in awake animals[24] (see also [22,23]). To reliably detect SPW-Rs (in an offline manner), we implemented a dual-criterion pipeline based on established routines[22,23], as follows. First, the instantaneous amplitude of the 80–250 Hz ripple-band signal was calculated as the absolute value of its Hilbert transform (*hilbert* function), followed by z-scoring using the mean and standard deviation (SD) of the entire envelope timeseries. Candidate ripple events were identified as periods where the z-scored envelope exceeded a high threshold of 4 SD for a minimum duration of 15 ms. The start and end times of each candidate event were defined by the points where the z-scored envelope crossed a lower threshold of 2 SD. Secondly, to ensure that the detected ripples were part of a complete SPW-R complex, each candidate ripple was validated by the presence of a coincident sharp-wave component (a brief, large deflection in the 5–40 Hz signal). To establish a robust detection threshold uninfluenced by the events themselves, we estimated the background SD of the sharp-wave band signal by temporarily clipping the values above 4 SD. For each candidate ripple, the corresponding window of the non-clipped sharp-wave band signal was extracted, and the event was validated only if the peak absolute amplitude of the sharp wave exceeded 2.5 background SDs. Note that while the recordings targeted the proximal *stratum radiatum*, sharp-wave deflections can appear negative or positive depending on the precise electrode depth relative to the CA1 dipole (*stratum radiatum* vs. *pyramidale*); this is why the sharp-wave component detection was performed using the absolute amplitude. Upon crossing this threshold, a duration criterion was enforced: we identified the zero-crossings flanking the peak of the sharp wave and required the duration between them to be between 20 ms and 400 ms. Candidate ripples failing either the amplitude or duration criteria for the associated sharp wave were excluded. All parameter values used in this routine were adopted from the corresponding studies[22,23].

For each validated SPW-R, a set of key parameters was extracted. (I) Ripple duration: Defined by the boundaries from the low ripple threshold (z=2; see previous paragraph). (II) Ripple amplitude: Defined as the peak amplitude of the envelope; quantified using the z-scored envelope as well as the raw envelope (µV). (III) Sharp-wave amplitude: Defined as the peak absolute amplitude of the sharp-wave deflection; quantified using the non-clipped sharp-wave band signal normalized to the background SD (z-scored) as well as the raw signal (µV). (IV) Intra-ripple frequency: Calculated as the mean of the instantaneous frequencies (inverse of the inter-peak intervals) of distinct oscillatory peaks within the ripple-band signal during the event. The peaks were identified using the *findpeaks* function. To assess peak oscillation speed, the maximum instantaneous frequency per event was also extracted and averaged

over events per mouse. Additionally, we computed (V) SPW-R occurrence rate, as the total number of validated SPW-Rs divided by the total recording duration (in seconds).

To investigate the temporal relationship between electrophysiological and population imaging events, LFP and two-photon imaging data were temporally aligned using synchronization pulses recorded in the acousto-optic deflector (AOD) log files. SPW-Rs were classified as coincident with an SE [(+SE)] if an SE was detected within a ±300 ms window around the ripple peak. SPW-Rs without an associated SE in this window were classified as non-coincident [(-SE)]. To analyze the population dynamics associated with these events, the fraction of active cells Φ(t) was extracted within a ±3.5 s window centered on each ripple peak. To account for differences in baseline activity across mice, these extracted Φ(t) traces were z-scored using a robust normalization procedure, as follows. For each mouse, we first calculated the mean and standard deviation (SD) of the population activity during the baseline period (-3.5 to -1.5 s) for each individual event. We then determined the median of these event-specific means and SDs to establish a robust session baseline mean and SD. Finally, all extracted Φ(t) traces for that mouse were z-scored using these statistics. For quantification, we calculated the maximum and mean (averaged over ±0.5 s) z-scored activity for (+SE) and (-SE) events.

To ensure the reliability of the analysis, LFP recordings were visually inspected for signal quality. One mouse was excluded from the SPW-R analysis due to a poor signal-to-noise ratio in the ripple band that precluded reliable event isolation and validation. The experimenters were blind to the experimental groups during the entire analysis.

- **Long-term synaptic depression induction *ex vivo***

The protocol for inducing NMDAR-dependent LTD in hippocampal slices from adult mice was adapted from [25]. Mice (n=6 for Ctrl-Ab, and n=7 for GluN1-Ab) were deeply anesthetized using isoflurane and the brain was quickly removed into ice-cold protective artificial cerebrospinal fluid (paCSF, in mM: 95 N-Methyl-D-Glucamine, 30 NaHCO3, 2.5 KCl, 1.25 NaH2PO4, 10 MgSO4, 0.5 CaCl2, 20 HEPES, 25 glucose, 2 thiourea, 5 Na-ascorbate, 3 Na-pyruvate, 12N-acetylcysteine, adjusted to pH7.3 and 300–310mOsmol, saturated with Carbogen). Transverse, 400µm-thick hippocampal slices (n = 6 mice/16 slices for Ctrl-Ab; n = 7 mice/21 slices for GluN1-Ab) were prepared in ice-cold paCSF on a vibratome (VT 1200S, Leica, Wetzlar, Germany) and transferred for warm recovery in paCSF at 33°C for 12 min. Thereafter, slices were transferred for recovery (at least 1h) at room temperature in aCSF$^+$ (containing in mM: 125 NaCl, 25 NaHCO3, 2.5 KCl, 1.25 NaH2PO4, 1 MgCl2, 2 CaCl2, 25 glucose, 2 thiourea, 5 Na-ascorbate, 3 Na-pyruvate, 12 N-acetylcysteine, adjusted to pH 7.3 and an osmolarity of 300–310 mOsmol, and saturated with Carbogen). For recordings, slices were transferred in a custom-made interface-like recording chamber under continuous perfusion (3 mL/min, Ismatec, Wertheim, Germany) with 30°C aCSF containing in mM 125 NaCl, 4.5 KCl, 25 NaHCO3, 1.25 NaH2PO4, 2 MgSO4, 3 CaCl2, 10 glucose, saturated with Carbogen.
We were blinded to the experimental treatment of the animals during recording and data analysis. CA1-PNs were visually identified using a microscope (Examine.Z1, Zeiss, Jena, Germany) equipped with differential interference contrast optics. Recording and stimulation pipettes were pulled using a P-87 horizontal pipette puller (Sutter Instruments, Novato, CA, USA) from thick-walled borosilicate glass (0.86x1.50, Science Products, Kamenz, Germany) and were filled with aCSF. The field excitatory postsynaptic potential (fEPSP) was recorded and digitized with a MultiClamp 700B amplifier (Molecular Devices, Sunnyvale, CA, USA), and an

Axon Digidata 1550B digitizer (Molecular Devices, Sunnyvale, CA, USA), respectively. Signals were low-pass filtered at 2kHz and digitized at 20kHz.
Stimulation of the Schaffer collaterals (SC) was performed with an aCSF-filled micropipette acting as a monopolar stimulation electrode that was placed 300µm away from the recording electrode. Stimulation was applied through a constant current stimulation unit (DS3, zigitimer). The LTD Stimulation protocol was conducted using the following steps. First, an input/output curve of field excitatory postsynaptic potential (fEPSP) responses to incremental SC stimulation was measured in the CA1 *stratum radiatum* by increasing stimulation strength from 20µA to 150µA delivered at 0.1Hz. Slices in which fEPSP slope changes >10% during the baseline recording, as well as slices which did not generate a fEPSP amplitude of at least 1mV were discarded. Stimulation strength was adjusted for ~50% (40–60%) of maximal fEPSC slope (mV/ms). LTD was induced after a baseline period of 20 minutes (stimulation at 0.05Hz) by three rounds of low-frequency stimulation (LFS) of 1500 pulses at 2Hz, with an interval of 10 minutes between each round [25]. This protocol results in an NMDAR-dependent (non-selective to NMDAR-subunit) LTD that persists in aged mice. After LTD inductions fEPSP slope was recorded for a further 40 minutes at 0.1Hz. Analysis of recorded signals was performed in Clampfit 10.5 (Molecular Devices, Sunnyvale, CA, USA).

- **Properties of putative neuron activity**

For each PN, we quantified the temporally local irregularity level of its CaT onsets using CV2, as a local and relatively rate-independent measure of spike-time irregularity [26]: $\mathrm{CV2} = \frac{2}{\mathrm{K}-1}\sum_{k=1}^{\mathrm{K}-1}\frac{|\mathrm{ICI}_{k+1}-\mathrm{ICI}_{k}|}{|\mathrm{ICI}_{k+1}+\mathrm{ICI}_{k}|}$, where ICI_k and ICI_{k+1} are the *k*th and (*k+1*)th inter-CaT intervals (ICIs) of the cell, and K is the total number of its ICIs. The global irregularity of each PN was computed using the coefficient of variation as $\mathrm{CV} = \sigma_{\mathrm{ICI}}/\mu_{\mathrm{ICI}}$, where σ_{ICI} and μ_{ICI} are the standard deviation and mean of the PN's ICIs. As illustrated in **Supplementary Fig. 2C**, to determine whether global irregularity of a PN is significantly beyond chance-level, we compared its empirical CV value ($\mathrm{CV}_{\mathrm{emp}}$) to those of its surrogate event trains ($\mathrm{CV}_{\mathrm{sh}}$), generated by shuffling its CaT onsets (uniform distribution, 500 times). This randomization kept the mean CaT frequency of the cell unchanged, while disrupting the precise timing of its CaTs. We then determined the 1st (δ) and 99th (Ψ) percentiles of its 500 $\mathrm{CV}_{\mathrm{sh}}$ values. Having repeated these steps for all PNs to determine the cell-specific δ and Ψ values, the population-level CV thresholds ($\mathrm{Thr}_{\mathrm{Low}}$ and $\mathrm{Thr}_{\mathrm{High}}$) were then computed as the median of these values , separately for 1st and 99th percentiles (i.e. δs and Ψs). For each FOV, these two population-level thresholds, derived from randomized surrogate data, were used to test whether a PN's $\mathrm{CV}_{\mathrm{emp}}$ is significantly different from the typical range expected based on its firing rate, relative to the population baseline: $\mathrm{CV}_{\mathrm{emp}}$ was considered significantly low (resp. high) if its smaller (resp. bigger) than $\mathrm{Thr}_{\mathrm{Low}}$ (resp. $\mathrm{Thr}_{\mathrm{High}}$). Using this approach, the significance of empirical CV2 values were also determined. Additionally, for each PN, we estimated its ICI correlation (ρICI), as the correlation of consecutive ICIs using Spearman's rank-order correlation of order one [27]. To achieve more robust results, PNs with less than ten ICIs were excluded for these measures.

- **Functional pairwise correlations**

To compute the pairwise correlation between neural activity of PNs (functional connectivity) we used three measures: 1) spike-time tiling coefficient (STTC), (2) Pearson correlation coefficient (ρCC), and (3) partial correlation coefficient (PCC). For two spike (here, CaT event) trains A and B, STTC is defined as [28]: $\mathrm{STTC} = \frac{1}{2}\left(\frac{P_A-T_B}{1-P_AT_B}+\frac{P_B-T_A}{1-P_BT_A}\right)$, where P_A is the proportion

of spikes (here, events) from A which lie within ±Δt (tiling window) of any spike from B, and T_A is the fraction of the total recording time which is covered by the tiling windows of A; P_B and T_B are similarly defined [28]. STTC is largely robust against firing rate levels and changes [28]. STTCs derived from measured data were compared to those from event-time exchanging (ETE) surrogate data, generated by randomly exchanging the CaT onsets across all network PNs, thereby preserving both the mean CaT frequency of each PN and the sum network activity per time-point (**Supplementary Fig. 3A**). This randomization was performed 500 times. For each pair, using its surrogates, we determined the significance of its empirical STTC (95th percentile), as illustrated in **Supplementary Fig. 3B**. For the tiling window, we set 2xΔt = 2, 4, 8, 12, 24, 48, or 96 frames, where 1 frame is ~100 ms. For ρCC, the event trains of A and B were first convolved with a Gaussian kernel with a defined standard deviation (Sigma), followed by computing the Pearson correlation coefficient between these smoothed activity traces [17,29,30]. For Sigma, we used 2, 4, 8, 12, 24, 48, or 96 frames. Using similar randomization approach as for STTC, the significance of the empirical ρCC indices were assessed (500 times shuffling, 99th percentile). Note that both STTC and ρCC between a pair of PNs can captures not only their direct coupling but also any indirect coupling induced by, for example, a third PN that sends input to both. Hence, to obtain an estimate potentially reflecting more direct pairwise interactions, we also utilized the Partial Correlation Coefficient (PCC); similarly to previous work [31,32]. PCC measures the linear correlation between the activity traces of two PNs after statistically accounting for (i.e., regressing out) the linear influence attributable to the activity of all other simultaneously recorded PNs, thereby attempting to isolate the direct functional relationship between the pair of interest. We computed PCC by adapting FARCI toolbox [32]. The partial correlation PCC_{ij} between the *i*th and *j*th PNs was computed as $\mathrm{PCC}_{ij} = -\phi_{ij}/\sqrt{\phi_{ii}\phi_{jj}}$, where the precision matrix $\phi = \Sigma^{-1}$, and Σ is NxN covariance matrix of neuronal activity among N neurons calculated based on the smoothed traces of CaT-onset trains. For smoothing, we convolved each train with a Gaussian kernel with a Sigma of 2, 4, 8, 12, 24, 48, or 96 frames. All these functional coupling were computed for all possible PN pairs (using custom-written or the adapted Matlab code) yielding symmetric NxN matrices, with values range from -1 and +1 which indicate perfect negative and positive correlations, respectively.

- **Functional neuronal assembly**

To detect PN ensembles we subjected the pairwise correlation matrices (see above) to the eigendecomposition clustering method, by adapting FluoroSNNAP toolbox [30]. Briefly, this method decomposes a given similarity matrix (here, STTC matrix or ρCC matrix) into a set of eigenvalues and eigenvectors. The number of significantly large eigenvalues determines the number of neuronal ensembles, and their corresponding eigenvectors contain the information about ensemble structure (i.e. the set of neurons belonging to each cluster). We used the same ETE surrogate data for testing the statistical significance (α = 5%) of the eigenvalues. This procedure enabled us to identify the ensembles of PNs which exhibited co-activity beyond chance level, while allowing for potential overlap between them. The contribution strength of each PN to its affiliated neuronal ensemble was quantified by its participation index (PI). For more details about this clustering method and its mathematical description see [33].

- **Functional network topology**

To determine the properties of functional network topology (connectivity motifs) we applied the complex network analysis [34] to thresholded STTC and PCC matrices. To this end, for each

FOV, we first binarized its empirical symmetric STTC (or PCC) matrix using the 95th percentile value of its ETE surrogate data. This was followed by subjecting the resulted binary (unweighted) and undirected matrix as the input to the functions implemented in the MATLAB Brain Connectivity Toolbox [34], where each PN was considered as a node and the edge (connection) between each pair of nodes was determined by their functional coupling (STTC or PCC). We quantified five topology metrics mainly relating to the clustering and centrality features of the network: I) node degree (ND), measuring the number of connections each PN has with other PNs, II) clustering coefficient (CC), measuring the likelihood that the neighbors of a given PN are also interconnected, III) eigenvector centrality (EVC), measuring the importance of a node in a network based on its connections to other highly central nodes, IV) betweenness centrality (BWC), measuring the extent to which a PN acts as a go-between for other PNs, and V) small-worldness (SW), measuring the balance between topologically local clustering and global connectivity in the network. To this end, we quantified I)-III) using *degrees_und*, *clustering_coef_bu*, and *eigenvector_centrality_und* functions, respectively. For BWC, we applied *betweenness_bin* function to the output of *weight_conversion* function implementing 'lengths' conversion method. For SW, we first obtained characteristic path length (CPL) by computing the distance matrix (*distance_bin* function) and using it as the input to *charpath* function without including distances on the main diagonal and infinite distances. CC and CPL values were normalized by dividing them by corresponding "null" values, which were determined by generating 100 synthetic random networks (*makerandCIJ_und* function), computing the same parameters at each iteration, and then averaging them per parameter. Finally, we computed SW by dividing the normalized CC by the normalized CPL. To account for the different number of nodes (N) across networks, we divided the BWCs by [(N-1)*(N-2)]. We also computed the normalized ND by dividing the absolute ND by the network size (N-1).

- **Quantification of network hubness**

To specifically quantify the presence and prominence of functional network hubs, i.e. nodes with disproportionately high connectivity or centrality, we applied two metrics to the distributions of node degree and betweenness centrality computed for each network. 1) Gini Coefficient: It measures the inequality of a distribution, and is adapted from economics[35] to assess network hubness here; for previous applications of this measure to neural data see [17,36]. The Gini coefficient for a given distribution is determined using the Lorenz curve. This curve plots the cumulative proportion of a variable (e.g., node degree) against the cumulative proportion of the population (e.g. PNs) possessing that variable, when the population is rank-ordered from lowest to highest value. A diagonal line represents perfect equality, where each member of the population holds an equal share of the total variable. The Gini coefficient quantifies the deviation of the Lorenz curve from this line of equality, measuring the overall inequality of the distribution. It ranges from 0 (perfect equality, where all members have the same value) to 1 (maximum inequality, where one member holds the entire amount of the variable). We calculated the Gini coefficient for the node degree (ND) distribution and the betweenness centrality (BWC) distribution of all PNs within each network graph. A higher Gini coefficient indicates a greater concentration of connectivity or centrality among a smaller fraction of nodes, signifying a more hub-dominated network structure. 2) Hub-Enrichment Ratio: To further assess the prominence of nodes in the upper tail of the distributions, we defined a hub-enrichment ratio as the ratio of the 75th percentile to the 50th percentile (i.e. median) of the ND distribution and, separately, the BWC distribution. A ratio notably greater than 1 indicates that the top 25% of nodes are disproportionately more connected or central

compared to that of the typical (median) node, providing direct evidence for the presence of highly influential hubs. These two metrics provide complementary, normalization-independent assessments of the extent to which network connectivity and centrality are concentrated in hub nodes.

- **Network activity properties**

For each FOV, we first computed network activity timeseries. To this end, in the CaT train of each PN, we set $\pm\omega t$ frames around each CaT to 1 whereby accounting for some temporal jitter in the detection of CaT-onsets (ωt was set to 1). This was followed by computing the mean across the resulting CaT vectors of all individual PNs to obtain the empirical fraction of active cells per frame $\Phi(t)$ [17]. To quantify the variability (fluctuations) in network activity we calculated the coefficient of variation of $\Phi(t)$. To assess the rhythmicity of network activity, we calculated the power spectral density (PSD) of $\Phi(t)$ using Welch's method (MATLAB *pwelch()* function) with 20 s Hamming windows, 75% overlap. The PSD of each FOV was normalized to its total PSD to obtain a more robust measure of oscillatory bands when averaging over mice per group. To detect synchronous events (SEs) as the significant network co-activation periods, we randomly shuffled CaT onsets of all PNs (uniform distribution; 1000 times), computed the surrogate $\Phi(t)$ (as above), and defined the 99.99th percentile of all surrogate $\Phi(t)$ as the threshold for SE detection. The SE threshold was determined separately for each FOV, to account for different mean CaT frequencies. We then considered any frame with an empirical $\Phi(t)$ exceeding the threshold as belonging to an SE. In the resulting binary SE vectors, $0\rightarrow1$ transitions were defined as SE onsets and $1\rightarrow0$ transitions as SE offsets. Using the binary SE vectors, we extracted following parameters: (1) the SE occurrence rate as the number of SEs divided by the total available recording time, (2) the relative time the network spent in SEs, (3) the average SE duration (offset *minus* onset *plus* 1frame), (4) SE size as the fraction of PNs which were active in at least one frame of a given SE. In addition, we computed the synchronization capacity of each PN as the fraction of SEs in which it participated, divided by its CaT frequency (in [1/s]) to control for different PN activity levels. Moreover, as a direct quantification of network's baseline activity level, we calculated the median of $\Phi(t)$ values that fell below the 3.25%; a conservative threshold derived from the minimum value of SE-thresholds across all mice.

- **Similarity of spatiotemporal patterns**

To investigate the similarity of SE spatiotemporal patterns, we represented each SE period as a binary spatial pattern (vector) of active and inactive PNs of size Nx1, where N is the number of PNs in FOV. We then quantified the similarity between each two patterns Pat_i and Pat_j using the so-called matching index[17,37]: $\mathrm{MI}_{ij} = \frac{|\mathrm{Pat}_i \cap \mathrm{Pat}_j|}{|\mathrm{Pat}_i|+|\mathrm{Pat}_j|}$. MI ranges from 0 (no similarity) to 1 (perfect similarity) and approximates the number of shared active PNs (i.e. common ones) between pattern pairs. For each pattern pair, we determined the significance of its empirical MI (99th percentile) using the surrogate data obtained by randomly shuffling the values within binary patterns (500 shuffles). In addition, to assess the patterns similarity as a function of SE size, we thresholded SEs based on their size, and recomputed the measure between the remaining SEs. For this, we used three thresholds obtained by calculating the quartiles of SE-detection thresholds (see above) of all FOVs: Q1≈3.9, Q2≈4.3, Q3≈4.9%. This is equivalent to using the same thresholds for all mice. To assess whether our findings in this analysis is specific to SE patterns or extends to the broader network activity, we divided the entire recording time

to non-overlapping bins, converted them to binary vectors, and computed their similarity using the same procedure (250 shuffles, 99th percentile). For the binning, we used windows of size 10, 20, 30, 40, or 50 frames. For more robust results, in addition to silent bins, the patterns including less than five active PNs were also excluded.

- **Inter-neuronal transmission metrics**

To investigate the effect of GluN1-Ab on inter-neuronal signal transmission [38–40], we considered each PN as a transmitter (PN_T, T: transmitter) and any other PN as a potential receiver (PN_R, T: transmitter). Based on the reconstructed binary CaT-onset trains of PNs (for brevity, we here refer to each CaT of PN_T as tCAT and of PN_R as rCaT), we approximated the reliability, efficacy, and latency of CaT transmission for each PN pair ($PN_T \rightarrow PN_R$) within a specified transmission time window (W_{TR}) after each tCaT. The reliability quantifies the proportion of tCaTs that successfully transmitted to PN_R within W_{TR}. It is defined as the ratio of tCaTs that have at least one corresponding rCaT to the total number of tCaTs. To avoid double (or multiple) counting, each rCaT is only considered for the closest tCaT within W_{TR}. The reliability ranges from 0 to 1, where 1 indicates 100% success rate for transmission (of note, the corresponding transmission failure can be determined as: 1 *minus* Reliability). The efficacy is computed as the total number of rCaTs occurring within the W_{TR} after each tCaT divided by the total number of tCaTs. Each rCaT is only counted once, even if it falls within the window of multiple tCaTs, to avoid double counting. The efficacy ranges from 0 to potentially greater than 1, depending on the specific neural activity patterns. The Latency refers to the average time delay between a tCaT and the corresponding rCaT within W_{TR}. The average latency is computed across all successful transmissions, with each rCaT only being counted once, by considering its latency to the closest tCaT. For the transmission window, we set W_{TR} = 3, 6, or 12 frames (1 frame $\approx$ 100 ms), consistent with the timescale of SE durations in our data and of plausible short-term synaptic plasticity [41]. For each FOV, this analysis provided us by a generally asymmetric NxN transmission matrix per parameter, where N is the number of PNs in FOV; note that the transmission properties of $PN_x \rightarrow PN_y$ channel is generally different from $PN_y \rightarrow PN_x$. We defined the reliability of each PN (as transmitter) by computing the median over its corresponding row, i.e. over its outgoing channels to all other PNs (as receivers), in the corresponding matrix. The efficacy (using median) and latency (using mean) of each PN were quantified similarly. We considered the PNs with the empirical reliability and efficacy levels bigger than 95th, and the empirical latencies smaller than 5th, of their corresponding ETE surrogate data as significant, separately. When reporting the reliability and efficacy of each PN, we subtracted the mean of its corresponding surrogate values to account for the potential differences in PN CaT frequencies.

- **Three bursting-neuron network model**

The relatively low temporal resolution of our two-photon imaging data (~100 ms) limits the ability to resolve the ability to resolve fast dynamics such as within-burst inter-spike intervals [17,21,42]. To demonstrate that this limitation does not affect the reliability of our key coupling results (STTC, PCC) at the correlation timescales used here (>100 ms), we used neuronal simulations. To this end, we employed a single-compartment model of hippocampal pyramidal cell (PC) [42], which can generate mixed firing patterns of single spikes and bursts of spikes, where an interplay of persistent Na^+ and M-type K^+ currents underlies its bursting behavior (mathematical description and parameter values are detailed in [42]). Based on this model, we built a three-neuron network model, with the following architecture: PC1 and PC2

are recurrently connected, and PC3 projects unidirectionally to both PC1 and PC2. For each PC, the synaptic conductance, $g_i(t)$, was modelled to instantaneously jump by G_{ij} upon arrival of a presynaptic spike (where, j is the index of presynaptic neuron, $G_{12} = G_{21} = 0.0025$, $G_{13} = G_{13} = 0.005$, in units of [mS/cm^2]), followed by an exponential decay with a synaptic decay time-constant of 5 ms (similarly to [43]). Each neuron also received an independent random background input ($\mu = 0.1$, $\sigma = 0.7$, in units of [μA /cm^2]) sufficient to make it fire spontaneously at a low rate. Simulations were run in Matlab using the Euler method at a time step of 0.05 ms resolution for 120 seconds, and different random seeds across 10 trials to generate variability. At each trial, each neuron exhibited a different spiking pattern. Spikes were detected when the membrane potential crossed a threshold of 0 mV. The detected spike trains were then binned (thus, downsampled) to two target resolutions: each bin containing at least one spike was assigned a value of 1, otherwise 0. Two final temporal resolutions were used: a high resolution of 1 kHz (1 ms bin size) and a low resolution of 10 Hz (100 ms, mimicking our imaging data). Note that at 10 Hz resolution, a value of 1 may encode may encode, similarly to our CaT event data, a single spike or multiple spikes (e.g. a burst). For simulations representing the direct correlations without PC3's influence, we removed connections from PC3 to PC1 and PC2 ($G_{13} = G_{23} = 0$), while keeping both the $G_{12} = G_{21} = 0.0025$ and the random seeds of background inputs the same as the corresponding full network trial. The simulated, downsampled binary event trains were used as the input to the coupling measures.

Quantification and statistical analysis

Statistical analyses were performed using OriginPro 2019 and MATLAB 2020a. Group assignment (Ctrl-Ab vs. GluN1-Ab) was performed in a pseudorandomized manner by an independent technician. We were blinded to the experimental treatment of the animals during both calcium imaging and electrophysiological recordings, as well as during all data analysis. All data are reported as mean ± standard error of the mean (SEM), unless stated otherwise. Specific details about sample sizes (n) are provided in each figure legend and **Supplementary Table 1** to ensure clarity and reproducibility. Group sizes were based on prior studies using similar analyses and were sufficient to detect group differences with comparable variance. The Shapiro–Wilk test was used to test for normality. Parametric testing procedures were applied for normally distributed data; otherwise, nonparametric tests were used. The *F*-test was used to assess homogeneity of variances; when variances were significantly different, Welch's correction was applied. Except for the Shapiro–Wilk test and F-test where p-values < 0.05 were considered statistically significant, the actual p-values are reported for all other tests within the figures and **Supplementary Table 1**. The skewness and Spearman's rank correlation coefficient were computed using *skewness* and *corr* Matlab functions. Curve permutation tests were performed by comparing the area under the curve (AUC) of the difference of the group-mean (or median) curves with those AUC values obtained after shuffling the individual curves across groups, repeated 1 million times; see Ref. [1] for more details. The distance of group-mean (or median) probability distribution of GluN1-Ab group and the equivalent distribution of Ctrl-Ab group (used as reference) was measured using the Kullback–Leibler divergence (KLD) metric. As with the curve permutation, the p-value of the empirical KLD (p-kld) was computed by comparing it to KLD values from 1 million shuffled iterations. This KLD permutation test was applied specifically on data showing no significant change in the mean or median, to test for the differences in distribution shape. For improved visualization only, the displayed distributions were smoothed using normal kernel density

estimation (*ksdensity* function in Matlab). Details of the applied statistical tests with the sample sizes are provided in **Supplementary Table 1**.

References

1 Ceanga M, Rahmati V, Haselmann H, Schmidl L, Hunter D, Brauer A-K *et al.* Human NMDAR autoantibodies disrupt excitatory-inhibitory balance, leading to hippocampal network hypersynchrony. *Cell Rep.* 2023; **42**: 113166.

2 Planagumà J, Leypoldt F, Mannara F, Gutiérrez-Cuesta J, Martín-García E, Aguilar E *et al.* Human N-methyl D-aspartate receptor antibodies alter memory and behaviour in mice. *Brain* 2015; **138**: 94–109.

3 Kreye J, Wenke NK, Chayka M, Leubner J, Murugan R, Maier N *et al.* Human cerebrospinal fluid monoclonal N-methyl-D-aspartate receptor autoantibodies are sufficient for encephalitis pathogenesis. *Brain.* 2016; **139**: 2641–2652.

4 Planagumà J, Haselmann H, Mannara F, Petit-Pedrol M, Grünewald B, Aguilar E *et al.* Ephrin-B2 prevents N-methyl-D-aspartate receptor antibody effects on memory and neuroplasticity. *Ann Neurol.* 2016; **80**: 388–400.

5 Tanaka K. Clinical profiles of Japanese patients with anti-NMDAR encephalitis and functional analysis of the anti-NMDAR antibodies. *Rinsho Shinkeigaku.* 2012; **52**: 985–987.

6 Zhang Q, Tanaka K, Sun P, Nakata M, Yamamoto R, Sakimura K *et al.* Suppression of synaptic plasticity by cerebrospinal fluid from anti-NMDA receptor encephalitis patients. *Neurobiol Dis.* 2012; **45**: 610–615.

7 Radosevic M, Planagumà J, Mannara F, Mellado A, Aguilar E, Sabater L *et al.* Allosteric Modulation of NMDARs Reverses Patients' Autoantibody Effects in Mice. *Neurol Neuroimmunol Neuroinflamm.* 2022; **9**: e1122.

8 Day C, Silva J-P, Munro R, Mullier B, André VM, Wolff C *et al.* Peptide-Purified Anti-N-methyl-D-aspartate Receptor (NMDAR) Autoantibodies Have Inhibitory Effect on Long-Term Synaptic Plasticity. *Pharmaceuticals (Basel).* 2024; **17**: 1643.

9 Luo Y, Yu Y, Zhang M, Fan N. GluN1 antibody causes behavioral deficits in prepulse inhibition and memory through CaMKIIβ signaling. *J Neuroimmunol.* 2022; **373**: 577998.

10 Wardemann H, Yurasov S, Schaefer A, Young JW, Meffre E, Nussenzweig MC. Predominant autoantibody production by early human B cell precursors. *Science.* 2003; **301**: 1374–1377.

11 Wenke NK, Kreye J, Andrzejak E, van Casteren A, Leubner J, Murgueitio MS *et al.* N-methyl-D-aspartate receptor dysfunction by unmutated human antibodies against the NR1 subunit. *Ann. Neurol.* 2019; **85**: 771–776.

12 Kreye J, Wright SK, van Casteren A, Stöffler L, Machule M-L, Reincke SM *et al.* Encephalitis patient-derived monoclonal GABAA receptor antibodies cause epileptic seizures. *The Journal of experimental medicine* 2021; **218**: e20210012.

13 Wright SK, Rosch RE, Wilson MA, Upadhya MA, Dhangar DR, Clarke-Bland C *et al.* Multimodal electrophysiological analyses reveal that reduced synaptic excitatory neurotransmission underlies seizures in a model of NMDAR antibody-mediated encephalitis. *Commun Biol.* 2021; **4**: 1106.

14 Mizrahi A, Crowley JC, Shtoyerman E, Katz LC. High-resolution in vivo imaging of hippocampal dendrites and spines. *J. Neurosci.* 2004; **24**: 3147–3151.

15 Stosiek C, Garaschuk O, Holthoff K, Konnerth A. In vivo two-photon calcium imaging of neuronal networks. *Proc. Natl Acad. Sci. USA.* 2003; **100**: 7319–7324.

16 Pnevmatikakis EA, Giovannucci A. NoRMCorre: An online algorithm for piecewise rigid motion correction of calcium imaging data. *J. Neurosci. Methods* 2017; **291**: 83–94.

17 Graf J, Rahmati V, Majoros M, Witte OW, Geis C, Kiebel SJ *et al.* Network instability dynamics drive a transient bursting period in the developing hippocampus in vivo. *eLife.* 2022; **11**: e82756.

18 Nimmerjahn A, Kirchhoff F, Kerr JND, Helmchen F. Sulforhodamine 101 as a specific marker of astroglia in the neocortex in vivo. *Nat. Methods.* 2004; **1**: 31–37.

19 Bezaire MJ, Soltesz I. Quantitative assessment of CA1 local circuits: knowledge base for interneuron-pyramidal cell connectivity. *Hippocampus.* 2013; **23**: 751–785.

20 Pelkey KA, Chittajallu R, Craig MT, Tricoire L, Wester JC, McBain CJ. Hippocampal GABAergic Inhibitory Interneurons. *Physiol Rev.* 2017; **97**: 1619–1747.

21 Rahmati V, Kirmse K, Holthoff K, Kiebel SJ. Ultra-fast accurate reconstruction of spiking activity from calcium imaging data. *J Neurophysiol.* 2018; **119**: 1863–1878.

22 Fernández-Ruiz A, Oliva A, Fermino de Oliveira E, Rocha-Almeida F, Tingley D, Buzsáki G. Long-duration hippocampal sharp wave ripples improve memory. *Science.* 2019; **364**: 1082–1086.

23 Oliva A, Fernández-Ruiz A, Buzsáki G, Berényi A. Role of Hippocampal CA2 Region in Triggering Sharp-Wave Ripples. *Neuron.* 2016; **91**: 1342–1355.

24 Stark E, Roux L, Eichler R, Senzai Y, Royer S, Buzsáki G. Pyramidal cell-interneuron interactions underlie hippocampal ripple oscillations. *Neuron.* 2014; **83**: 467–480.

25 Ahmed T, Sabanov V, D'Hooge R, Balschun D. An N-methyl-D-aspartate-receptor dependent, late-phase long-term depression in middle-aged mice identifies no GluN2-subunit bias. *Neuroscience.* 2011; **185**: 27–38.

26 Holt GR, Softky WR, Koch C, Douglas RJ. Comparison of discharge variability in vitro and in vivo in cat visual cortex neurons. *J Neurophysiol.* 1996; **75**: 1806–1814.

27 Farkhooi F, Strube-Bloss MF, Nawrot MP. Serial correlation in neural spike trains: experimental evidence, stochastic modeling, and single neuron variability. *Phys. Rev. E. Stat. Nonlin. Soft Matter Phys.* 2009; **79**: 21905.

28 Cutts CS, Eglen SJ. Detecting pairwise correlations in spike trains: an objective comparison of methods and application to the study of retinal waves. *J Neurosci.* 2014; **34**: 14288–14303.

29 Flossmann T, Kaas T, Rahmati V, Kiebel SJ, Witte OW, Holthoff K *et al.* Somatostatin Interneurons Promote Neuronal Synchrony in the Neonatal Hippocampus. *Cell Rep.* 2019; **26**: 3173–3182.e5.

30 Patel TP, Man K, Firestein BL, Meaney DF. Automated quantification of neuronal networks and single-cell calcium dynamics using calcium imaging. *J Neurosci Methods.* 2015; **243**: 26–38.

31 Ota K, Oisi Y, Suzuki T, Ikeda M, Ito Y, Ito T *et al.* Fast, cell-resolution, contiguous-wide two-photon imaging to reveal functional network architectures across multi-modal cortical areas. *Neuron.* 2021; **109**: 1810–1824.e9.

32 Meamardoost S, Bhattacharya M, Hwang EJ, Komiyama T, Mewes C, Wang L *et al.* FARCI: Fast and Robust Connectome Inference. *Brain Sci.* 2021; **11**: 1556.

33 Li X, Ouyang G, Usami A, Ikegaya Y, Sik A. Scale-free topology of the CA3 hippocampal network: a novel method to analyze functional neuronal assemblies. *Biophys J.* 2010; **98**: 1733–1741.

34 Rubinov M, Sporns O. Complex network measures of brain connectivity: uses and interpretations. *NeuroImage.* 2010; **52**: 1059–1069.

35 Sitthiyot T, Holasut K. A simple method for measuring inequality. *Humanit Soc Sci Commun.* 2020; **6**: 112.

36 Mizuseki K, Buzsáki G. Preconfigured, skewed distribution of firing rates in the hippocampus and entorhinal cortex. *Cell Rep.* 2013; **4**: 1010–1021.
37 Romano SA, Pietri T, Pérez-Schuster V, Jouary A, Haudrechy M, Sumbre G. Spontaneous neuronal network dynamics reveal circuit's functional adaptations for behavior. *Neuron.* 2015; **85**: 1070–1085.
38 Andrade-Talavera Y, Fisahn A, Rodríguez-Moreno A. Timing to be precise? An overview of spike timing-dependent plasticity, brain rhythmicity, and glial cells interplay within neuronal circuits. *Mol Psychiatry.* 2023; **28**: 2177–2188.
39 Ermentrout GB, Galán RF, Urban NN. Reliability, synchrony and noise. *Trends Neurosci.* 2008; **31**: 428–434.
40 Budak M, Zochowski M. Synaptic Failure Differentially Affects Pattern Formation in Heterogenous Networks. *Front Neural Circuits.* 2019; **13**: 31.
41 Rahmati V, Kirmse K, Holthoff K, Schwabe L, Kiebel SJ. Developmental Emergence of Sparse Coding: A Dynamic Systems Approach. *Sci Rep.* 2017; **7**: 13015.
42 Rahmati V, Kirmse K, Marković D, Holthoff K, Kiebel SJ. Inferring Neuronal Dynamics from Calcium Imaging Data Using Biophysical Models and Bayesian Inference. *PLoS Comput Biol.* 2016; **12**: e1004736.
43 Shriki O, Hansel D, Sompolinsky H. Rate models for conductance-based cortical neuronal networks. *Neural Comput.* 2003; **15**: 1809–1841.
