# Supplementary material for: Anti-NMDAR encephalitis impairs intrinsic hippocampal dynamics through neuronal hypercoupling, hub dominance, and aberrant ensembles
Source: Mol Psychiatry. 2026 Mar 31;31(8):4550–62. doi: 10.1038/s41380-026-03568-6 (PMC13364672; doi:10.1038/s41380-026-03568-6)
Supplement: Supplementary file 2 — Supplementary Table 1 [file 41380_2026_3568_MOESM2_ESM.pdf]

Related to	Descriptive statistics	Test(s)	Test statistics
**Supplementary Fig. 1**	**Group: mean ± SEM (no. of mice)**		
S1C (inset)			P = 0.9503
Total power (µV^2)	Ctrl-Ab: 0.88490 ± 0.19456 (n=9)	Two-sample t-test, two-tailed	t = 0.0633
	GluN1-Ab: 0.86954 ± 0.14719 (n=9)		df = 16
S1D			P = 0.4894
Bandpower (norm.)	Ctrl-Ab: 0.00189 ± 1.76588E-4 (n=9)	Mann-Whitney U-test (exact), two-tailed	Z = 0.70642
	GluN1-Ab: 0.00177 ± 2.88959E-4 (n=9)		U = 49
Bandpower (µV^2)			P = 0.6375
	Ctrl-Ab: 4.5217 ± 1.2479 (n=9)	Two-sample t-test, two-tailed	t = 0.4803
	GluN1-Ab: 3.8304e ± 0.71709 (n=9)		df = 16
Peak power (norm.)			P = 0.2224
	Ctrl-Ab: 0.00418 ± 6.48068E-4 (n=9)	Mann-Whitney U-test (exact), two-tailed	Z = 1.23623
	GluN1-Ab: 0.00395 ± 0.00138 (n=9)		U = 55
Peak power (µV^2)			P = 0.5457
	Ctrl-Ab: 10.5569 ± 3.73331 (n=9)	Mann-Whitney U-test (exact), two-tailed	Z = 0.61812
	GluN1-Ab: 8.31071 ± 2.75882 (n=9)		U = 48
Peak frequency (Hz)			P = 0.2667
	Ctrl-Ab: 1.62972 ± 0.03259 (n=9)	Mann-Whitney U-test (exact), two-tailed	Z = -1.1101
	GluN1-Ab: 1.7039 ± 0.05853 (n=9)		U = 27.5
**Figure 1**	**Group: mean ± SEM (no. of mice)**		
1E			
CaT frequency distributions [1/s]	Ctrl-Ab: (n=9)	Permutation test of KLD, one-tailed, 1 million shuffles	P = 0.038
	GluN1-Ab: (n=9)		
CaT frequency [1/s] (mean)			P = 0.1634
	Ctrl-Ab: 0.0556 ± 0.0029 (n=9)	Two-sample t-test, two-tailed	t = 1.4607
	GluN1-Ab: 0.0476 ± 0.0047 (n=9)		df = 16
1G (top)			P = 0.0320
CV of ICI (significantly high CVs, median)	Ctrl-Ab: 1.32 ± 0.0157 (n=9)	Two-sample t-test, two-tailed	t = -2.3486
	GluN1-Ab: 1.3862 ± 0.0234 events (n=9)		df = 16
1G (bottom)			P = 0.0214
CV2 of ICI (significantly low CV2s, mean)	Ctrl-Ab: 0.7793 ± 0.0058 (n=97)	Two-sample t-test, two-tailed	t = 2.5499
	GluN1-Ab: 0.7503 ± 0.0098 (n=9)		df = 16
1I (top)			P = 0.0255
correlation of CV and CV2 of cells	Ctrl-Ab: 0.1280 ± 0.0355 (n=9)	Two-sample t-test, two-tailed	t = 2.4623
	GluN1-Ab: -0.0453 ± 0.0608 (n=9)		df = 16
1I (bottom)			P = 0.01876
correlation of CV and median ICI of cells	Ctrl-Ab: -0.0519 ± 0.0463 (n=9)	Mann-Whitney U-test (exact), two-tailed	Z = 2.29586
	GluN1-Ab: -0.2844 ± 0.0646 (n=9)		U = 67
1J (top)			
summed power of 0.05-2 Hz band	Ctrl-Ab: (n=9)	Curve permuation, one-tailed, 1 million shuffles	P = 0.0157
	GluN1-Ab: (n=9)		
1J (bottom)			P = 0.04
bandpower of 0.05-1 Hz band	Ctrl-Ab: 0.1319 ± 0.0039 (n=9)	Mann-Whitney U-test (exact), two-tailed	Z = 2.03095
	GluN1-Ab: 0.1114 ± 0.0083 (n=9)		U = 64
1K			P = 0.03147
Φ variability (coefficient of variation)	Ctrl-Ab: 0.6758 ± 0.0322 (n=9)	Mann-Whitney U-test (exact), two-tailed	Z = -2.11925
	GluN1-Ab: 0.9529 ± 0.1395 (n=9)		U = 16
1L			P = 0.0315
SE rate [Hz]	Ctrl-Ab: 0.09468 ± 0.00651 (n=9)	Two-sample t-test, two-tailed	t = 2.3571
	GluN1-Ab: 0.07257 ± 0.00675 (n=9)		df = 16
1M			
SE-size distributions [%]	Ctrl-Ab: (n=9)	Permutation test of KLD, one-tailed, 1 million shuffles	P = 0.037
	GluN1-Ab: (n=9)		
1N (inset)			P = 0.01419
synchronization capacitiy (SC, mean)	Ctrl-Ab: 1.7192 ± 0.0836 (n=9)	Mann-Whitney U-test (exact), two-tailed	Z = -2.38416
	GluN1-Ab: 2.5773 ± 0.3679 (n=9)		U = 13
1O			P = 0.04
bandpower (<0.05 Hz)	Ctrl-Ab: 0.0019 ± 0.0002 (n=9)	Mann-Whitney U-test (exact), two-tailed	Z = -2.03095
	GluN1-Ab: 0.0029 ± 0.0004 (n=9)		U = 17
1P			P = 0.0251
non-SE Φ activity [%] (median)	Ctrl-Ab: 1.4261 ± 0.0755 (n=9)	Two-sample t-test, two-tailed	t = 2.4701
	GluN1-Ab: 1.0467 ± 0.1337 (n=9)		df = 16
**Supplementary Fig. 2**	**Group: mean ± SEM (no. of mice)**		
S2A			P = 0.3346
CV of ICI (mean)	Ctrl-Ab: 1.0745 ± 0.0261 (n=9)	Two-sample t-test, two-tailed	t = -0.9949
	GluN1-Ab: 1.1139 ± 0.0297 (n=9)		df = 16
S2B			P = 0.1666
CV2 of ICI (median)	Ctrl-Ab: 0.8563 ± 0.0086 (n=9)	Two-sample t-test, two-tailed	t = 1.4490
	GluN1-Ab: 0.8311 ± 0.0151 (n=9)		df = 16
S2D (top)			P = 0.0594
CV of ICI (significantly low CVs, mean)	Ctrl-Ab: 0.7531 ± 0.0106 (n=9)	Two-sample t-test, two-tailed	t = 2.0292
	GluN1-Ab: 0.7165 ± 0.0146 (n=9)		df = 16
S2D (bottom)			P = 0.2657
CV2 of ICI (significantly high CV2s, mean)	Ctrl-Ab: 1.2454 ± 0.0104 (n=9)	Two-sample t-test, two-tailed	t = -1.1561
	GluN1-Ab: 1.2631 ± 0.0106 (n=9)		df = 15
S2E (top)			P = 0.0295
correlation of CV and CaT frequency of cells	Ctrl-Ab: -0.1011 ± 0.0480 (n=9)	Two-sample t-test, two-tailed	t = -2.3893
	GluN1-Ab: 0.0885 ± 0.0632 (n=9)		df = 16
S2E (bottom)			P = 0.1143
correlation of CV2 and CaT frequency of cells	Ctrl-Ab: -0.6777 ± 0.0226 (n=9)	Two-sample t-test, two-tailed	t = -1.6702
	GluN1-Ab: -0.6137 ± 0.0309 (n=9)		df = 16
S2F (inset)			P = 0.0339
ρICI (positive values, mean)	Ctrl-Ab: 0.1455 ± 0.0066 (n=9)	Two-sample t-test, two-tailed	t = -2.3189
	GluN1-Ab: 0.1706 ± 0.0086 (n=9)		df = 16
S2G			
distributions of Φ [%]	Ctrl-Ab: (n=9)	Permutation test of KLD, one-tailed, 1 million shuffles	P = 0.005
	GluN1-Ab: (n=9)		
S2H (top)			P = 0.93143
SE-threshold [%]	Ctrl-Ab: 4.3004 ± 0.1436 (n=9)	Mann-Whitney U-test (exact), two-tailed	Z = -0.0883
	GluN1-Ab: 4.6016 ± 0.44 (n=9)		U = 39
S2H (bottom)			P = 0.3262
fraction of time spent in SEs [%]	Ctrl-Ab: 2.82 ± 0.27 (n=9)	Two-sample t-test, two-tailed	t = 1.01288
	GluN1-Ab: 2.38 ± 0.33 (n=9)		df = 16
S2I (top)			P = 0.47563
SE duration (mean) [s]	Ctrl-Ab: 0.5143 ± 0.0154 (n=9)	Two-sample t-test (Welch correction), two-tailed	t = -0.7377
	GluN1-Ab: 0.5411 ± 0.0329 (n=9)		df = 11.3578
S2I (bottom)			P = 0.9951
SE duration (range) [s]	Ctrl-Ab: 0.6896 ± 0.0362 (n=9)	Two-sample t-test (Welch correction), two-tailed	t = -0.0062
	GluN1-Ab: 0.6901 ± 0.0.0847 (n=9)		df = 10.8189
**Figure 2**	**Group: mean ± SEM (no. of mice)**		
2B (inset)			P = 0.02443
STTC of postive PN pairs (median)	Ctrl-Ab: 0.0135 ± 0.0006 (n=9)	Mann-Whitney U-test (exact), two-tailed	Z = -2.20755
	GluN1-Ab: 0.0191 ± 0.0036 (n=9)		U = 15
2C (inset)			P = 0.03147
STTC of significant PN pairs (median)	Ctrl-Ab: 0.0464 ± 0.0015 (n=9)	Mann-Whitney U-test (exact), tow-tailed	Z = -2.11925

	GluN1-Ab: 0.0664 ± 0.0101 (n=9)		U = 16
2E (top)<br>mean STTC of postive PN pairs	Ctrl-Ab: (n=9)<br>GluN1-Ab: (n=9)	Curve permuation, one-tailed, 1 million shuffles	P = 0.0116
2E (bottom)<br>Mean STTC of significant PN pairs	Ctrl-Ab: (n=9)<br>GluN1-Ab: (n=9)	Curve permuation, one-tailed, 1 million shuffles	P = 0.0045
2F<br>median STTC of significant PN pairs	Ctrl-Ab: (n=9)<br>GluN1-Ab: (n=9)	Curve permuation, one-tailed, 1 million shuffles	P = 0.0352
2G<br>mean PCC of all PN pairs	Ctrl-Ab: (n=9)<br>GluN1-Ab: (n=9)	Curve permuation, one-tailed, 1 million shuffles	P = 0.0315
**Supplementary Fig. 3**	**Group: mean ± SEM (no. of mice)**		
S3C<br>fraction of significant PN pairs [%]	Ctrl-Ab: (n=9)<br>GluN1-Ab: (n=9)	Curve permuation, one-tailed, 1 million shuffles	P = 0.1818
S3D<br>median STTC of positive PN pairs (AUC)	Ctrl-Ab: (n=9)<br>GluN1-Ab: (n=9)	Curve permuation, one-tailed, 1 million shuffles	P = 0.1381
At the first tiling-window (0.2 sec)<br>STTC (median)	Ctrl-Ab: 0.0135 ± 0.0006 (n=9)<br>GluN1-Ab: 0.0191 ± 0.0036 (n=9)	Mann-Whitney U-test (exact), two-tailed	P = 0.02443<br>Z = -2.20755<br>U = 15
At the second tiling-window (0.4 sec)<br>STTC (median)	Ctrl-Ab: 0.0177 ± 0.0008 (n=9)<br>GluN1-Ab: 0.0247 ± 0.0046 (n=9)	Mann-Whitney U-test (exact), two-tailed	P = 0.03998<br>Z = -2.03095<br>U = 17
S3E<br>mean ρCC of significant PN pairs<br>Over 0.2-0.8 sec time slaces	Ctrl-Ab: (n=9)<br>GluN1-Ab: (n=9)	Curve permuation, one-tailed, 1 million shuffles	P = 0.0373
Over all time slaces (0.2-9.6 sec)	Ctrl-Ab: (n=9)<br>GluN1-Ab: (n=9)	Curve permuation, one-tailed, 1 million shuffles	P = 0.0672
S3F<br>mean ρCC of significant PN pairs	Ctrl-Ab: (n=9)<br>GluN1-Ab: (n=9)	Curve permuation, one-tailed, 1 million shuffles	P = 0.007
S3G (left)<br>mean PCC of all PN pairs	Ctrl-Ab: (n=9)<br>GluN1-Ab: (n=9)	Curve permuation, one-tailed, 1 million shuffles	P = 0.039
S3G (right)<br>mean PCC of positive PN pairs	Ctrl-Ab: (n=9)<br>GluN1-Ab: (n=9)	Curve permuation, one-tailed, 1 million shuffles	P = 0.0138
S3K (left)<br>first bar vs second bar	ρCC (w/ PC3): 0.3898 ± 0.01609 (#Trials=10)<br>PCC (w/ PC3): 0.3688 ± 0.01678 (#Trials=10)	Paired sample t-test, two-tailed	P = 0.0024<br>t = 4.17288<br>df = 9
second bar vs third bar	PCC (w/ PC3): 0.3688 ± 0.01678 (#Trials=10)<br>ρCC (w/o PC3): 0.3541 ± 0.02472 (#Trials=10)	Paired sample t-test, two-tailed	P = 0.3537<br>t = -0.9779<br>df = 9
**Figure 3**	**Group: mean ± SEM (no. of mice)**		
3A (top)<br>median node degree, based on STTC	Ctrl-Ab: (n=9)<br>GluN1-Ab: (n=9)	Curve permuation, one-tailed, 1 million shuffles	P = 0.0526
3A (bottom)<br>median node degree, based on PCC	Ctrl-Ab: (n=9)<br>GluN1-Ab: (n=9)	Curve permuation, one-tailed, 1 million shuffles	P = 0.0232
3D (left,box plot)<br>Gini of node degree (timescale: 0.2 sec)	Ctrl-Ab: 0.1689 ± 0.00565 (n=9)<br>GluN1-Ab: 0.2238 ± 0.01964 (n=9)	Mann-Whitney U-test (exact), two-tailed	P = 0.0028<br>Z = -2.82567<br>U = 8
3D (right)<br>Gini of node degree (all timescale)	Ctrl-Ab: (n=9)<br>GluN1-Ab: (n=9)	Curve permuation, one-tailed, 1 million shuffles	P = 0.0068
3E (left,box plot)<br>Gini of betweenness (timescale: 0.2 sec)	Ctrl-Ab: 0.2954 ± 0.0091(n=9)<br>GluN1-Ab: 0.3628 ± 0.0207 (n=9)	Two-sample t-test, two-tailed	P = 0.0089<br>t = -2.97718<br>df = 16
3D (right)<br>Gini of betweenness (all timescale)	Ctrl-Ab: (n=9)<br>GluN1-Ab: (n=9)	Curve permuation, one-tailed, 1 million shuffles	P = 0.0080
3F (top)<br>median clustering coefficient, based on STTC	Ctrl-Ab: (n=9)<br>GluN1-Ab: (n=9)	Curve permuation, one-tailed, 1 million shuffles	P = 0.0113
3F (bottom)<br>median clustering coefficient, based on PCC	Ctrl-Ab: (n=9)<br>GluN1-Ab: (n=9)	Curve permuation, one-tailed, 1 million shuffles	P = 0.0032
3I (top)<br>Count	Ctrl-Ab: (n=9)<br>GluN1-Ab: (n=9)	Curve permuation, one-tailed, 1 million shuffles	P = 0.0160
3I (bottom)<br>Ratio	Ctrl-Ab: (n=9)<br>GluN1-Ab: (n=9)	Curve permuation, one-tailed, 1 million shuffles	P = 0.0864
3J (right, top)<br>median amplitude of all sEPSC [pA]	Ctrl-Ab: 18.8385 ± 1.6366 (n= 6 mice/11 cells)<br>GluN1-Ab: 14.6348 ± 1.0622 (n=6 mice/14 cells)	Two-sample t-test, two-tailed	P = 0.0352<br>t = 2.2377<br>df = 23
3J (right, bottom)<br>median amplitude of strong sEPSC [pA]	Ctrl-Ab: 82.7514 ± 21.4614 (n= 6 mice/11 cells)<br>GluN1-Ab: 47.9356 ± 6.8489 (n=6 mice/14 cells)	Mann-Whitney U-test (exact), two-tailed	P = 0.10632<br>Z = 1.61497<br>U = 107
3K (yellow-colored period)<br>fEPSP slope [rel.]	Ctrl-Ab: 6 mice, 16 slices<br>GluN1-Ab: 7 mice, 21 slices	Mixed-model analysis: group x time	ß = -0.000946<br>SE = 0.000244<br>P < 0.0001
**Supplementary Fig. 4**	**Group: mean ± SEM (no. of mice)**		
S4A (top)<br>total #connections, based on STTC	Ctrl-Ab: (n=9)<br>GluN1-Ab: (n=9)	Curve permuation, one-tailed, 1 million shuffles	P = 0.0374
S4A (bottom)<br>total #connections, based on PCC	Ctrl-Ab: (n=9)<br>GluN1-Ab: (n=9)	Curve permuation, one-tailed, 1 million shuffles	P = 0.0247
S4B (top)<br>mean norm. node degree, based on STTC	Ctrl-Ab: (n=9)<br>GluN1-Ab: (n=9)	Curve permuation, one-tailed, 1 million shuffles	P = 0.1170
S4B (bottom)<br>mean norm. node degree, based on PCC	Ctrl-Ab: (n=9)<br>GluN1-Ab: (n=9)	Curve permuation, one-tailed, 1 million shuffles	P = 0.1706
S4C (top)<br>Gini of node degree, based on STTC	Ctrl-Ab: (n=9)<br>GluN1-Ab: (n=9)	Curve permuation, one-tailed, 1 million shuffles	P =0.1239
S4C (bottom)<br>Gini of betweenness, based on STTC	Ctrl-Ab: (n=9)<br>GluN1-Ab: (n=9)	Curve permuation, one-tailed, 1 million shuffles	P = 0.0154

S4D (top)<br>Hub enrichment ratio of node degree,<br>based on STTC	Ctrl-Ab: (n=9)<br>GluN1-Ab: (n=9)	Curve permuation, one-tailed, 1 million shuffles	P = 0.0515
S4D (bottom)<br>Hub enrichment ratio of node degree,<br>based on PCC	Ctrl-Ab: (n=9)<br>GluN1-Ab: (n=9)	Curve permuation, one-tailed, 1 million shuffles	P = 0.0044
S4E (top)<br>Hub enrichment ratio of betweenness,<br>based on STTC	Ctrl-Ab: (n=9)<br>GluN1-Ab: (n=9)	Curve permuation, one-tailed, 1 million shuffles	P = 0.0035
S4E (bottom)<br>Hub enrichment ratio of betweenness,<br>based on PCC	Ctrl-Ab: (n=9)<br>GluN1-Ab: (n=9)	Curve permuation, one-tailed, 1 million shuffles	P = 0.0087
S4F (top, left)<br>median small-worldness, based on STTC	Ctrl-Ab: (n=9)<br>GluN1-Ab: (n=9)	Curve permuation, one-tailed, 1 million shuffles	P = 0.0081
S4F (top, middle)<br>mean betweenness, based on STTC	Ctrl-Ab: (n=9)<br>GluN1-Ab: (n=9)	Curve permuation, one-tailed, 1 million shuffles	P = 0.0358
S4F (top, right)<br>mean eigenvector centrality, based on STTC	Ctrl-Ab: (n=9)<br>GluN1-Ab: (n=9)	Curve permuation, one-tailed, 1 million shuffles	P = 0.0384
S4F (bottom, left)<br>median small-worldness, based on PCC	Ctrl-Ab: (n=9)<br>GluN1-Ab: (n=9)	Curve permuation, one-tailed, 1 million shuffles	P = 0.0032
S4F (bottom, middle)<br>mean betweenness, based on PCC	Ctrl-Ab: (n=9)<br>GluN1-Ab: (n=9)	Curve permuation, one-tailed, 1 million shuffles	P = 0.0300
S4F (bottom, right)<br>mean eigenvector centrality, based on PCC	Ctrl-Ab: (n=9)<br>GluN1-Ab: (n=9)	Curve permuation, one-tailed, 1 million shuffles	P = 0.0329
S4H (left)<br>Count	Ctrl-Ab: (n=9)<br>GluN1-Ab: (n=9)	Curve permuation, one-tailed, 1 million shuffles	P = 0.0129
S4H (right)<br>Ratio	Ctrl-Ab: (n=9)<br>GluN1-Ab: (n=9)	Curve permuation, one-tailed, 1 million shuffles	P = 0.1567
**Figure 4**	**Group: mean ± SEM (no. of mice)**		
4B (right, top)<br>mean reliability of significant PNs	Ctrl-Ab: 0.01198 ± 0.0007 (n=9)<br>GluN1-Ab: 0.0179 ± 0.0029 (n=9)	Mann-Whitney U-test (exact), tow-tailed	P = 0.01876<br>Z = -2.29586<br>U = 14
4B (right, bottom)<br>mean latency of significant PNs	Ctrl-Ab: 0.2601 ± 0.0043 (n=9)<br>GluN1-Ab: 0.23356 ± 0.0902 (n=9)	Mann-Whitney U-test (exact), tow-tailed	P = 0.01061<br>Z = 2.47246<br>U = 69
4C (top)<br>mean reliability of significant PNs	Ctrl-Ab: (n=9)<br>GluN1-Ab: (n=9)	Curve permuation, one-tailed, 1 million shuffles	P = 0.006
4C (bottom)<br>mean latency of significant PNs	Ctrl-Ab: (n=9)<br>GluN1-Ab: (n=9)	Curve permuation, one-tailed, 1 million shuffles	P = 0.0059
4F (top, right)<br>median MI of significant SE pattern pairs	Ctrl-Ab: 0.2188 ± 0.0098 (n=9)<br>GluN1-Ab: 0.2786 ± 0.0302 (n=9)	Mann-Whitney U-test (exact), two-tailed	P = 0.05944<br>Z = -1.8553<br>U = 19
4F (top, left)<br>Skewness of significant MI values	Ctrl-Ab: 1.3393 ± 0.1694 (n=9)<br>GluN1-Ab: 0.9835 ± 0.1563 (n=9)	Mann-Whitney U-test (exact), two-tailed	P = 0.03998<br>Z = 2.03095<br>U = 64
4F (bottom)<br>median of significant, thresholded SE patterns	Ctrl-Ab: (n=9)<br>GluN1-Ab: (n=9)	Curve permuation, one-tailed, 1 million shuffles	P = 0.0210
4G (inset)<br>meidan MI of significant binned-pattern pairs	Ctrl-Ab: 0.1673 ± 0.0051 (n=9)<br>GluN1-Ab: 0.2068 ± 0.0208 (n=9)	Mann-Whitney U-test (exact), two-tailed	P = 0.03731<br>Z = -2.03727<br>U = 17
4H<br>median MI of significant binned-pattern pairs<br>(for statistics related to the star see 4G [inset])	Ctrl-Ab: (n=9)<br>GluN1-Ab: (n=9)	Curve permuation, one-tailed, 1 million shuffles	P = 0.3005
4I<br>mean STTC of ensemble PNs	Ctrl-Ab: (n=9)<br>GluN1-Ab: (n=9)	Curve permuation, one-tailed, 1 million shuffles	P = 0.0892
At tiling window of 1.2 sec	Ctrl-Ab: 0.0853 ± 0.0029 (n=4)<br>GluN1-Ab: 0.1270 ± 0.0109 (n=6)	Two-sample t-test (Welch correction), two-tailed	P = 0.01132<br>t = -3.68507<br>df = 5.68051
4J<br>mean participation index of ensemble PNs	Ctrl-Ab: (n=9)<br>GluN1-Ab: (n=9)	Curve permuation, one-tailed, 1 million shuffles	P = 0.1482
At tiling window of 1.2 sec	Ctrl-Ab: 0.09286 ± 0.00399 (n=4)<br>GluN1-Ab: 0.13351 ± 0.0106 (n=6)	Two-sample t-test (Welch correction), two-tailed	P = 0.0106<br>t = -3.58748<br>df = 6.30823
4K<br>nean reliability of ensemble PNs	Ctrl-Ab: (n=9)<br>GluN1-Ab: (n=9)	Curve permuation, one-tailed, 1 million shuffles	P = 0.1344
At tiling window of 1.2 sec	Ctrl-Ab: 0.04175 ± 0.00407 (n=4)<br>GluN1-Ab: 0.07071 ± 0.00764 (n=6)	Two-sample t-test (Welch correction), two-tailed	P = 0.0117<br>t = -3.34454<br>df = 7.26136
**Supplementary Fig. 5**	**Group: mean ± SEM (no. of mice)**		
S5A<br>mean efficacy of significant PNs	Ctrl-Ab: 0.0112 ± 0.0006 (n=9)<br>GluN1-Ab: 0.017 ± 0.0031 (n=9)	Mann-Whitney U-test (exact), two-tailed	P = 0.02443<br>Z = -2.20755<br>U = 15
S5B<br>mean efficacy of significant PNs	Ctrl-Ab: (n=9)<br>GluN1-Ab: (n=9)	Curve permuation, one-tailed, 1 million shuffles	P = 0.0122
S5C (left)<br>fraction of PNs with significant reliability	Ctrl-Ab: (n=9)<br>GluN1-Ab: (n=9)	Curve permuation, one-tailed, 1 million shuffles	P = 0.1358
S5C (middle)<br>fraction of PNs with significant efficacy	Ctrl-Ab: (n=9)<br>GluN1-Ab: (n=9)	Curve permuation, one-tailed, 1 million shuffles	P = 0.1513
S5C (right)<br>fraction of PNs with significant latency	Ctrl-Ab: (n=9)<br>GluN1-Ab: (n=9)	Curve permuation, one-tailed, 1 million shuffles	P = 0.3974
S5D (left)<br>median MI of all SE pattern pairs	Ctrl-Ab: (n=9)<br>GluN1-Ab: (n=9)	Curve permuation, one-tailed, 1 million shuffles	P = 0.0254
S5D (right)			

fraction of SE pattern pairs with significant MI	Ctrl-Ab: (n=9)<br>GluN1-Ab: (n=9)	Curve permuation, one-tailed, 1 million shuffles	P = 0.0901
S5E (left)<br>median MI of all binned-pattern pairs	Ctrl-Ab: (n=9)<br>GluN1-Ab: (n=9)	Curve permuation, one-tailed, 1 million shuffles	P = 0.1564
S5E (right)<br>fraction of binned pattern pairs with significant MI	Ctrl-Ab: (n=9)<br>GluN1-Ab: (n=9)	Curve permuation, one-tailed, 1 million shuffles	P = 0.0279
S5F<br>mean PI variability of ensemble PNs	Ctrl-Ab: (n=9)<br>GluN1-Ab: (n=9)	Curve permuation, one-tailed, 1 million shuffles	P = 0.0628
At tiling window of 1.2 sec	Ctrl-Ab: 0.3766 ± 0.0364 (n=4)<br>GluN1-Ab: 0.5138 ± 0.0211 (n=6)	Mann-Whitney U-test (exact), two-tailed	P = 0.01905<br>Z = -2.23861<br>U = 1
S5G (Left)<br>mean efficacy of ensemble PNs	Ctrl-Ab: (n=9)<br>GluN1-Ab: (n=9)	Curve permuation, one-tailed, 1 million shuffles	P = 0.1276
At tiling window of 1.2 sec	Ctrl-Ab: 0.04165 ± 0.00375 (n=4)<br>GluN1-Ab: 0.0706 ± 0.00772 (n=6)	Two-sample t-test (Welch correction), two-tailed	P = 0.0119<br>t = -3.3723<br>df = 6.9889
S5G (right)<br>mean latency of ensemble PNs	Ctrl-Ab: (n=9)<br>GluN1-Ab: (n=9)	Curve permuation, one-tailed, 1 million shuffles	P = 0.2388
**Supplementary Fig. 6**	**Group: mean ± SEM (no. of mice)**		
S6B (left)<br>Mean Intra-Ripple frequency	Ctrl-Ab: 140.32 ± 1.2911 (n=9)<br>GluN1-Ab: 149.25 ± 3.1151 (n=8)	Mann-Whitney U-test (exact), two-tailed	P = 0.02065<br>Z = -2.26129<br>U = 12
S6B (right)<br>Maximum Intra-Ripple frequency	Ctrl-Ab: 159.83 ± 1.6542 (n=9)<br>GluN1-Ab: 169.35 ± 3.4103 (n=8)	Mann-Whitney U-test (exact), two-tailed	P = 0.01522<br>Z = -2.35751<br>U = 11
S6C<br>Ooccurrence rate of SPW-Rs	Ctrl-Ab: 0.095128 ± 0.020281 (n=9)<br>GluN1-Ab: 0.12458 ± 0.026018 (n=8)	Two-sample t-test, two-tailed	P = 0.38053<br>t = -0.90352<br>df = 15
S6D<br>Ripple duration	Ctrl-Ab: 25.886 ± 1.1766 (n=9)<br>GluN1-Ab: 28.038 ± 0.98725 (n=8)	Two-sample t-test, two-tailed	P = 0.18762<br>t = -1.38063<br>df = 15
S6E (left)<br>Ripple amplitude (zscore)	Ctrl-Ab: 8.3404 ± 0.51882 (n=9)<br>GluN1-Ab: 9.0522 ± 0.50619 (n=8)	Mann-Whitney U-test (exact), two-tailed	P = 0.67297<br>Z = -0.43301<br>U = 31
S6E (right)<br>Ripple amplitude (mV)	Ctrl-Ab: 0.10113 ± 0.018624 (n=9)<br>GluN1-Ab: 0.12872 ± 0.022319 (n=8)	Two-sample t-test, two-tailed	P = 0.35407<br>t = -0.95632<br>df = 15
S6F (left)<br>SPW amplitude (zscore)	Ctrl-Ab: 4.1669 ± 0.2398 (n=9)<br>GluN1-Ab: 4.7769 ± 0.2809 (n=8)	Two-sample t-test, two-tailed	P = 0.11729<br>t = -1,66189<br>df = 15
S6F (right)<br>SPW amplitude (mV)	Ctrl-Ab: 0.15306 ± 0.022735 (n=9)<br>GluN1-Ab: 0.1955 ± 0.025094 (n=8)	Mann-Whitney U-test (exact), two-tailed	P = 0.54142<br>Z = -0.62546<br>U = 29
S6G<br>Fraction of ripples involved SE (%)	Ctrl-Ab: 47.41 ± 4.4864 (n=9)<br>GluN1-Ab: 34.32 ± 7.048 (n=8)	Two-sample t-test, two-tailed	P = 0.12956<br>t = 1.60399<br>df = 15
S6I: maximum active cells fraction (zscore)<br>(left): (+SE)	Ctrl-Ab: 4.8861 ± 0.16998 (n=9)<br>GluN1-Ab: 7.655 ± 1.2555 (n=8)	Mann-Whitney U-test (exact), two-tailed	P = 0.00058<br>Z = -3.12731<br>U = 31
(right): (-SE)	Ctrl-Ab: 0.51118 ± 0.12863 (n=9)<br>GluN1-Ab: 0.59927 ± 0.069059 (n=8)	Mann-Whitney U-test (exact), two-tailed	P = 0.27659<br>Z = -1.10659<br>U = 24
S6J: mean of active cells fraction (zscore)<br>(left): (+SE)	Ctrl-Ab: 2.0579 ± 0.093411 (n=9)<br>GluN1-Ab: 3.1393 ± 0.56781 (n=8)	Mann-Whitney U-test (exact), two-tailed	P = 0.0079<br>Z = -2.54996<br>U = 9
(right): (-SE)	Ctrl-Ab: 0.050727 ± 0.097164 (n=9)<br>GluN1-Ab: 0.16582 ± 0.05615 (n=8)	Mann-Whitney U-test (exact), two-tailed	P = 0.32127<br>Z = -1.01036<br>U = 25
